# Identification of lipidomic profiles associated with drug-resistant prostate cancer cells

**DOI:** 10.1186/s12944-021-01437-5

**Published:** 2021-02-17

**Authors:** Lishann M. Ingram, Morgan C. Finnerty, Maryam Mansoura, Chau-Wen Chou, Brian S. Cummings

**Affiliations:** 1 Pharmaceutical and Biomedical Sciences, 450 College of Pharmacy South, University of Georgia, Athens, GA 30602 USA; 2 Proteomics and Mass Spectrometry Facility (PAMS), Department of Chemistry, University of Georgia, Athens, GA USA; 3 Interdisciplinary Toxicology Program, University of Georgia, Athens, GA USA

**Keywords:** Drug resistance, Lipids, Lipidomics, Lipid metabolism, Lipid species, Mass spectrometry, Metastasis, Prostate Cancer, Prostate

## Abstract

**Background:**

The association of circulating lipids with clinical outcomes of drug-resistant castration-resistant prostate cancer (DR-CRPC) is not fully understood. While it is known that increases in select lipids correlate to decreased survival, neither the mechanisms mediating these alterations nor the correlation of resistance to drug treatments is well characterized.

**Methods:**

This gap-in-knowledge was addressed using in vitro models of non-cancerous, hormone-sensitive, CRPC and drug-resistant cell lines combined with quantitative LC-ESI-Orbitrap-MS (LC-ESI-MS/MS) lipidomic analysis and subsequent analysis such as Metaboanalyst and Lipid Pathway Enrichment Analysis (LIPEA).

**Results:**

Several lipid regulatory pathways were identified that are associated with Docetaxel resistance in prostate cancer (PCa). These included those controlling glycerophospholipid metabolism, sphingolipid signaling and ferroptosis. In total, 7460 features were identified as being dysregulated between the cell lines studied, and 21 lipid species were significantly altered in drug-resistant cell lines as compared to nonresistant cell lines. Docetaxel resistance cells (PC3-Rx and DU145-DR) had higher levels of phosphatidylcholine (PC), oxidized lipid species, phosphatidylethanolamine (PE), and sphingomyelin (SM) as compared to parent control cells (PC-3 and DU-145). Alterations were also identified in the levels of phosphatidic acid (PA) and diacylglyceride (DAG), whose levels are regulated by Lipin (LPIN), a phosphatidic acid phosphatase that converts PA to DAG. Data derived from cBioPortal demonstrated a population of PCa patients expressing mutations aligning with amplification of LPIN1, LPIN2 and LPIN3 genes. Lipin amplification in these genes correlated to decreased survival in these patients. Lipin-1 mRNA expression also showed a similar trend in PCa patient data. Lipin-1, but not Lipin-2 or − 3, was detected in several prostate cancer cells, and was increased in 22RV1 and PC-3 cell lines. The increased expression of Lipin-1 in these cells correlated with the level of PA.

**Conclusion:**

These data identify lipids whose levels may correlate to Docetaxel sensitivity and progression of PCa. The data also suggest a correlation between the expression of Lipin-1 in cells and patients with regards to prostate cancer cell aggressiveness and patient survivability. Ultimately, these data may be useful for identifying markers of lethal and/or metastatic prostate cancer.

**Supplementary Information:**

The online version contains supplementary material available at 10.1186/s12944-021-01437-5.

## Introduction

Drug resistance is a major obstacle for the development of PCa treatments. Although reports show that taxane-mediated microtubule stabilization differentially affects the androgen receptor, Docetaxel, a first-line chemotherapy for metastatic CRPC, has a known associated mechanism of resistance [1, 2]. The actual mechanisms linking changes in lipids levels to the generation of drug resistance in prostate cancer cells are unclear. This is despite several studies correlating unbalanced cellular lipid composition and quantity to altered cellular functions that contribute to drug resistance [3].

Cancer cells regulate their cellular lipids in a multifaceted process. Extensive studies provide strong evidence for the reprogramming of lipid metabolism in cancer [4–6]. Many of these studies are fueled by advances in mass spectrometry allowing for enhanced analysis of changes in the cellular or blood lipidome. While these studies have identified specific lipids, they have been hampered by the fact that changes in these lipids have not been put into context with changes in the regulation of overall lipid metabolism pathways. Further, changes in these lipids have not been correlated to changes in androgen or drug resistance. One reason for this gap-in-knowledge is that, unlike genomic and proteomics, analysis tools allowing for assessment of lipid regulation pathways in tandem with lipidomic outcomes are not common. Recent advances have resulted in the development of such tools, such as the Lipid Pathway Enrichment Analysis (LIPEA) a web-based tool for over-representation analysis of lipid signatures detection and enriched in biological pathways [7]. However, this approach has only seen limited application and has not been applied to studies in cancer cells.

PCa cells, in part, obtain and employ lipids to meet increasing energy demands for cell proliferation in a nutrient-deprived tumor microenvironment [8–14]. Many of the mechanisms mediating changes in lipid profiles during this process are not well understood. The present study used LIPEA, in conjunction with data derived from MetaboAnalyst to identify the underlying regulatory lipid pathways associated with Docetaxel resistance in PCa. Furthermore, lipidomic changes were the focal point in multiple models, including non-cancerous, hormone-sensitive, CRPC and Docetaxel resistant cell lines. The pathway analysis was further validated using LC-ESI-MS/MS. These studies present a comprehensive identification of differences in lipid profiles in drug resistant prostate cancer cell lines, castration-resistant and hormone-sensitive cells and further suggest novel links between select lipid metabolism enzymes and prostate cancer aggressiveness and patient survivorship.

## Materials and methods

### Cell culture

Cell lines used in this study were purchased from ATCC and include PC-3, LNCaP, 22RV-1, DU-145, RWPE1 and PNT2 (Manassas, VA, USA). The Docetaxel resistant human DU145-DR, PC3-Rx as well as a separate batch of DU-145 and PC-3 cell lines were provided by Dr. Begona Mellado’s laboratory in the Medical Oncology Department, Hospital Clinic de Barcelona, Spain. A second Docetaxel resistant human PC3-Rx cell line was provided by Prof. Lisa G Horvath’s laboratory in the Garvan Institute of Medical Research (Darlinghurst, Australia). Cell supplements, including primary cell culture media and antibiotics, were purchased from ATCC (Manassas, VA, USA). Standard cell culture media were purchased from Corning Inc. (Corning, NY, USA). Human prostate cancer cells were cultured in 10% FBS (Seradigm, Radnor, PA, USA) and 1% penicillin/streptomycin supplemented RPMI-1640, respectively. Cell lines were all incubated in 95% humidity and 5% CO_2_ at 37 °C. Resistance was maintained in Docetaxel resistant cell lines by dosing cells with a range of nM concentrations of Docetaxel at every 2nd and 4th passage. MTT assays were conducted to generate dose response curves to check Docetaxel resistance levels.

### Bligh-Dyer lipid extraction

Cells were washed twice, harvested in 1x phosphate buffered saline (PBS), and subsequently centrifuged. Phospholipids from cells were then immediately extracted using both chloroform and methanol/water according to Bligh and Dyer method [15]. Cell lines were suspended in 3 mL of each methanol/water and chloroform. Tubes were vortexed for 30 s and sat under the hood on ice for 10 min, followed by centrifugation (300 x *g*; 5 min). The bottom-most layer of chloroform was then transferred to a new test tube and spiked with a mix of commercialized SPLASH Lipidomix internal standards (Avanti Polar Lipids, Inc., Alabaster, Alabama, USA). SPLASH Lipidomix Mass Spec standards include all major lipid classes at ratios similar to those of human plasma. This extraction procedure was repeated three times and the chloroform layers from each extraction were combined. Collected chloroform layers were dried under nitrogen, reconstituted with 50 μL of methanol: chloroform (3:1 v/v), and stored at 80 °C until analysis.

### Liquid phosphorus assay

Lipid content was quantified by determining the level of inorganic phosphorus using the Bartlett Assay [16]. Sulfuric acid 400 μL (5 M) was added to lipid extracts (10 μL) in a glass test tube and heated at 180–200 °C for 1 h. H_2_O_2_ (100 μL of 30% v/v) was then added while vortexing and the extract heated at 180–200 °C for 1.5 h. Reagent (4.6 mL of 1.1 g ammonium molybdate tetrahydrate in 12.5 mL sulfuric acid and 500 mL ddH_2_O) was added followed by vortexing. Then, 100 μL of 15% ascorbic acid (v/v) was added, followed by further vortexing. The solution was then heated for 7–10 min at 100 °C. A 150 μL aliquot was used to measure the absorbance at 830 nm.

### ESI-MS/MS analysis of cells

Samples were run in triplicate (*n* = 3) and the most abundant species were defined as the core lipid pool [17]. Lipid extracts (500 pmol/μL) were prepared by reconstitution in chloroform: methanol (2:1, v/v). ESI-MS was performed as previously described [18–20] using an LCQ Deca ion-mass spectrometer (LCQ Finnigan mass spectrometer (Thermo Fisher-Fenning Institute, CA, USA)) with a nitrogen drying gas flow-rate of 81/min at 350 °C and a nebulizer pressure of 30 psi. The scanning range was from 200 to 1000 *m/z* on 5 μL of the samples scanned in the positive and negative mode for 2.5 min with a mobile phase of acetonitrile; methanol; water (2:3:1) in 0.1% ammonium formate.

### NanoHRLC-LTQ-Orbitrap MS

Lipid extracts were also analyzed using a Thermo-Fisher LTQ Orbitrap Elite Mass Spectrometer coupled with a Proxeon Easy NanoLC system (Waltham, MA, USA) located at Proteomics and Mass Spectrometry Facility (University of Georgia, Athens, GA, USA) [21].

Personnel running samples were blinded to sample conditions. Mass spectra were acquired in the positive ion mode. Mass spectrometry specifications for lipid extracts were as follows: spray voltage: 1.7–1.8 kV, ion transfer tube (or capillary); temperature: 200 °C, respectfully. Full scan, data-dependent MS/MS (top8-ddMS2), were collected at m/z 150–2000 (350–1800), corresponding to the mass range of most expected cellular lipids. Each run was externally calibrated before beginning to allow for LC-HRMS analysis at 120,000 resolution (at m/z 400) and MS/MS at 15,000-30,000.

Lipids were separated on a nanoC18 column (length, 130 mm; i.d., 100 μm; particle size, 5 μm; pore size, 150 Å; max flow rate, 500 nL/min; packing material, Bruker Micron Magic 18). Mobile phase A was 0.1% formic acid/water; mobile phase B was 0.1% formic acid/acetonitrile. 1.5 μL of each sample was injected for analysis. A constant flow rate of 450–500 nL/min was applied to perform a gradient profiling with the following proportional change of solvent A (v/v): 0–2 min at 98% A, in 40 min from 100% A to 5% A, kept at 5% A for 10 min, then lowered to 50% A in 10 min. A wash run with a high-organic gradient and an equilibrium run were inserted between runs to minimize carryover. The autosampler temperature was maintained at 7 °C for all experiments. Solvent extraction blanks and samples were jointly analyzed throughout each batch (10–15 samples).

### Data processing

Full scan raw data files were acquired from Xcalibur™ (Thermo Fisher Scientific (Waltham, MA, USA)), centroided and converted to a usable format (mzXML) using MSConvert. Data processing and peak area integration were performed using MZmine [22], and XCMS [23], resulting in a feature intensity table. Feature tables and MS/MS data were placed into a directory for each substrate analyzed. Each folder contained each sample type, feature tables end in “pos.csv” for positive mode. Features were identified using LipidMatch [24, 25]. Peak areas were normalized to a mixture of deuterium labeled internal standards for each sample (SPLASH® LIPIDOMIX® Mass Spec Standard).

### Multivariate statistical analysis of cells

Multivariate principal component analysis (PCA) was performed using MetaboAnalyst 3.0 [26]. Automatic peak detection and spectrum deconvolution was performed using a peak width set to 0.5. Analysis parameters consisted of interquartile range filtering and sum normalization with no removal of outliers from the dataset. Volcano plot analysis was used to select features, and MS/MS analysis was used to further identify features. Significance for volcano plot analysis was determined based on a fold-change threshold of 2.00 and *P* 0.05. Following identification of each feature, the parent lipid level was normalized using total ion count, and the change in the relative abundance of that phospholipid species compared to its control was determined. This is a standard method for lipidomic analysis as reported in previous studies [20, 27]. A schematic diagram of analytical strategy and sequence of analysis using the different LC/MS instruments and software is presented in Supplemental Figure 1.

### Pathway enrichment analysis

Pathway enrichment analysis of metabolites was performed using LIPEA software [7]. Total lipid compounds from all the pathways were extracted and the over-representation analysis (ORA) starts in parallel for each pathway. The server computes the Benjamini and Bonferroni *P*-value corrections when all the ORA analyses are completed. Once finished, the server returneda list of enriched pathways sorted by *P*-value. The final results were presented in an interactive table. The significance of the pathway fit is calculated with comparison to Fisher’s exact test performed on numerous permutations of random features within the total feature list. Hierarchical clustering of these data identified differentially expressed lipid pathways from the set of lipids identified in this study. The module predicted biological activity directly from the mass spectrometric peak list data and implemented the *mummichog* algorithm, which was cross referenced with the KEGG database. Biochemical pathways were derived from transformed KEGG IDs, using the internal mapping process (connected to Swiss Lipids, Lipid Maps, HMDB and KEGG databases) [7]. Columns represent individual sample types; rows refer to distinct metabolites, lipids and genes. Shades of green represent low levels and shades of red represent high levels (*P* < 0.05).

### Alterations in LPIN genes in human patients

Sixteen prostate studies were identified in the cBioPortal for Cancer Genomics (Table 2) [28, 29]. The three lipin genes (LPIN1, LPIN2, and LPIN3) were queried. Alterations in lipin genes within four types of prostate cancer were generated under the Cancer Types Summary through the selection of Cancer Type Detailed and Alteration Frequency. The overall survivability in prostate cancer patients with or without lipin gene alteration was determined using the Comparison/Survival tab and selecting survival. Within GEPIA 2, Single Gene Analysis and Boxplots were selected [30]. Variation in expression levels of lipin genes were observed in patients with and without prostate cancer (PRAD). The method for differential analysis was a one-way ANOVA, using disease state (Tumor or Normal) as the variable for calculating differential expression. The expression data are first log_2_(TPM + 1) transformed for differential analysis and the log_2_FC is defined as median (Tumor) - median (Normal). Genes with higher |log_2_FC| values and lower q values than pre-set thresholds are considered differentially expressed genes [28]. Multiple Gene Analysis and Correlation Analysis were selected. Pair-wise gene expression correlations, using the Pearson method, of the lipin genes were analyzed based on TCGA and prostate GTEx databases.

### Western blotting

Anti-rabbit primary antibodies for Lipin-1 (Abcam, ab181389) and Lipin-2 were purchased from Abcam (Cambridge, UK). The anti-rabbit primary antibody for Lipin-3 was purchased from Biorbyt (Cambridge, UK). The anti-mouse primary antibody for GAPDH was purchased from Sigma-Aldrich (St. Louis, MO). The anti-mouse and anti-rabbit secondary antibodies were purchased from Promega (Madison, WI) (Supplemental Table 2). HepG2 cells were purchased from Sigma-Aldrich (St. Louis, MO) and used the same standard cell culture media and care as the prostate cell lines. Whole-cell lysates were prepared using RIPA buffer (50 mM Tris-HCl [pH 7.4], 1 M NaCl, 1% NP-40, 0.5% sodium deoxycholate, 0.1% SDS, and ddH_2_O) and 1% protease inhibitor cocktail from Cell Signaling Technology (Danvers, MA). Lysates were then vortexed and frozen at − 80 °C. Samples were run in triplicate (*n* = 3). Proteins (5 μg) were resolved by 10% SDS-PAGE and transferred onto nitrocellulose membranes with a pore size of 0.45 μm (Thermo Scientific). Membranes were blocked with 5% milk powder (AppliChem) in 1X TBST for 1 h at room temperature. Incubations with primary antibodies at 4 °C overnight were followed by incubations with the appropriate secondary antibodies at room temperature for 1 h and detection by Pierce™ SuperSignal™ West Pico PLUS Chemiluminescent Substrate from Thermo Scientific (Waltham, MA). Images were captured using the FluorChem® HD2 Imager from Alpha Innotech (San Leandro, CA). Densitometry to quantify protein expression was performed using the software FluorChem® HD2 software from Alpha Innotech (San Leandro, CA). Lipin expression was normalized to GAPDH expression.

### Statistical analyses

All statistical analyses were compiled using GraphPad Prism for windows version 8.2.1 (GraphPad Software, Inc., La Jolla, CA, USA). For all analyses, the experimental unit was individual samples obtained from a minimum of 4 (*n* = 4) groups were assessed. One passage of cells was equivalent to one group of samples (n). The effect of multiple testing was controlled for by measuring the statistical significance of each association using both the *P*-value and the *q*-value. Using FDR of *q* < 0.05, the *q* value quantifies significance in terms of the false discovery rate (FDR) rather than the false positive rate and forms a measure of how likely a particular *P*-value is to represent a genuine association. For all analyses, significance was set at *P* 0.05 where data are expressed as mean ± SEM based on t-test for pairwise analysis and/or ANOVA analysis (with Kruskal-Wallis post hoc test).

## Results

### Comprehensive LC-ESI-MS/MS analysis between PCa cells

**Fig. 1:**
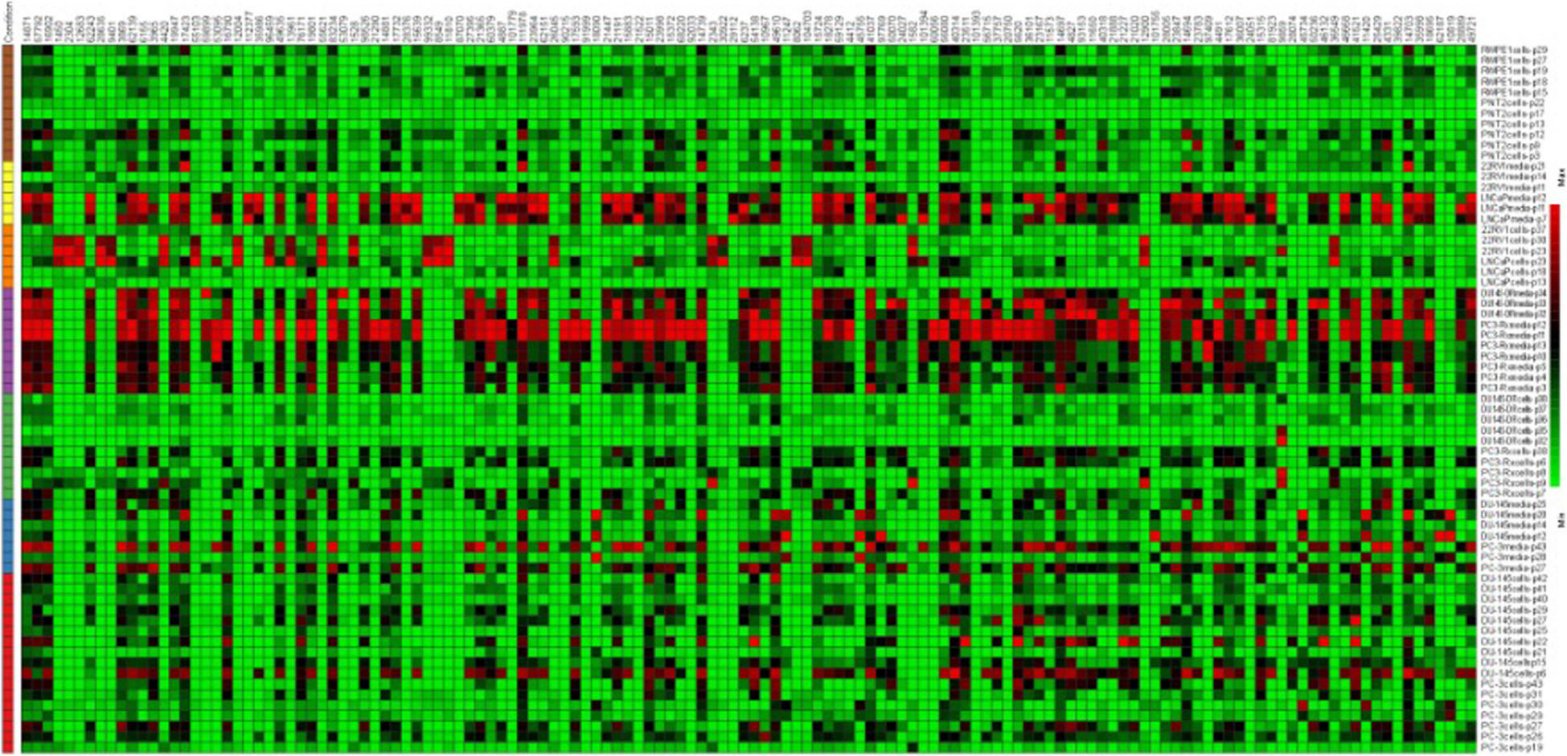
Heat map of differentially altered metabolites associated with non-cancerous (PNT2 and RWPE1), hormone-sensitive (LNCaP and 22RV1), castration-resistant (PC-3 and DU-145) and Docetaxel resistant (PC3-Rx and DU145-DR) prostate cell lines and media as determined by LC-ESI-MS/MS. Data were derived from a minimum of 3 extractions from 3 different passages per cell line

**Fig. 2:**
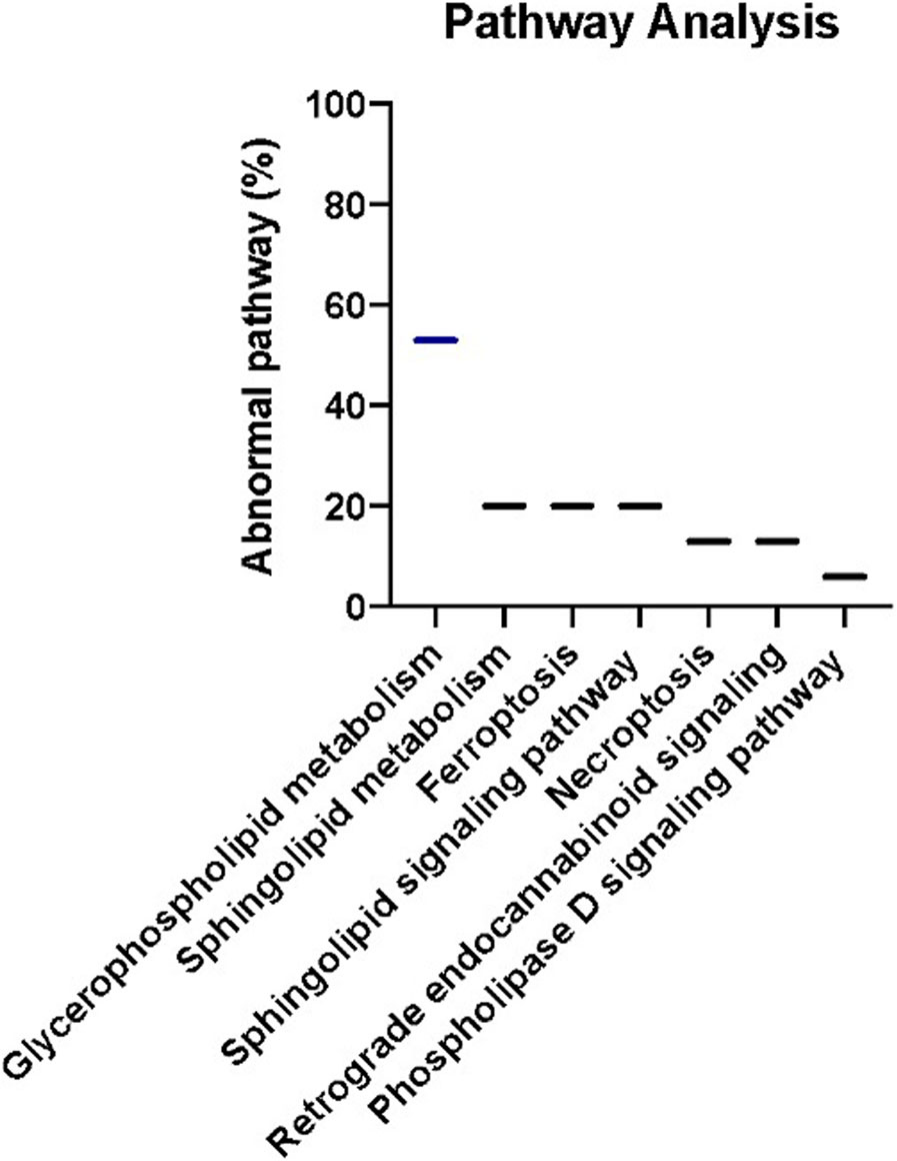
LIPEA pathway analysis. Identification of lipids pathways enriched in prostate cancer cells (LNCaP, 22RV1, PC-3, DU-145, PC3-Rx and DU145-DR) as compared to non-cancerous cells (PNT2 and RWPE1). Data were derived from a minimum of 3 extractions from 3 different passages, where increases in lipids were mapped to genes identified in the KEGG database

While it is well known that the development and progression of prostate cancer is associated with abnormal changes in lipids, the association of these lipids with specific signaling pathways has not received as much attention. Further, even less attention has been given to identifying these lipids, or the signaling pathways involved, with the development of drug resistance. This gap-in-knowledge was addressed by constructing a heat map using MS Peak to Pathway-MetaboAnalyst [26] comparing changes in lipid levels, as determined by LC-ESI-MS/MS, between non-cancerous (PNT2 and RWPE1), hormone-sensitive (LNCaP and 22RV1), castration-resistant (PC-3 and DU-145) and Docetaxel resistant (PC3-Rx and DU145-DR) prostate cell lines (Fig. 1). Pathway analysis was also conducted using the MS Peaks to Pathway Activities module from MetaboAnalyst, which generated a heat map-specific pathway visualization [31, 32]. This analysis resulted in several common dysregulated metabolic pathways including glycerophospholipid and sphingolipid metabolism. These data were confirmed with a LIPEA analysis, which showed that glycerophospholipid and sphingolipid metabolism were highly ranked and significantly associated with the set of lipids identified in this study. Other pathways identified included ferroptosis (20%) and choline metabolism in cancer (20%) (Fig. 2, Supplemental Table 1) [7].

**Fig. 3:**
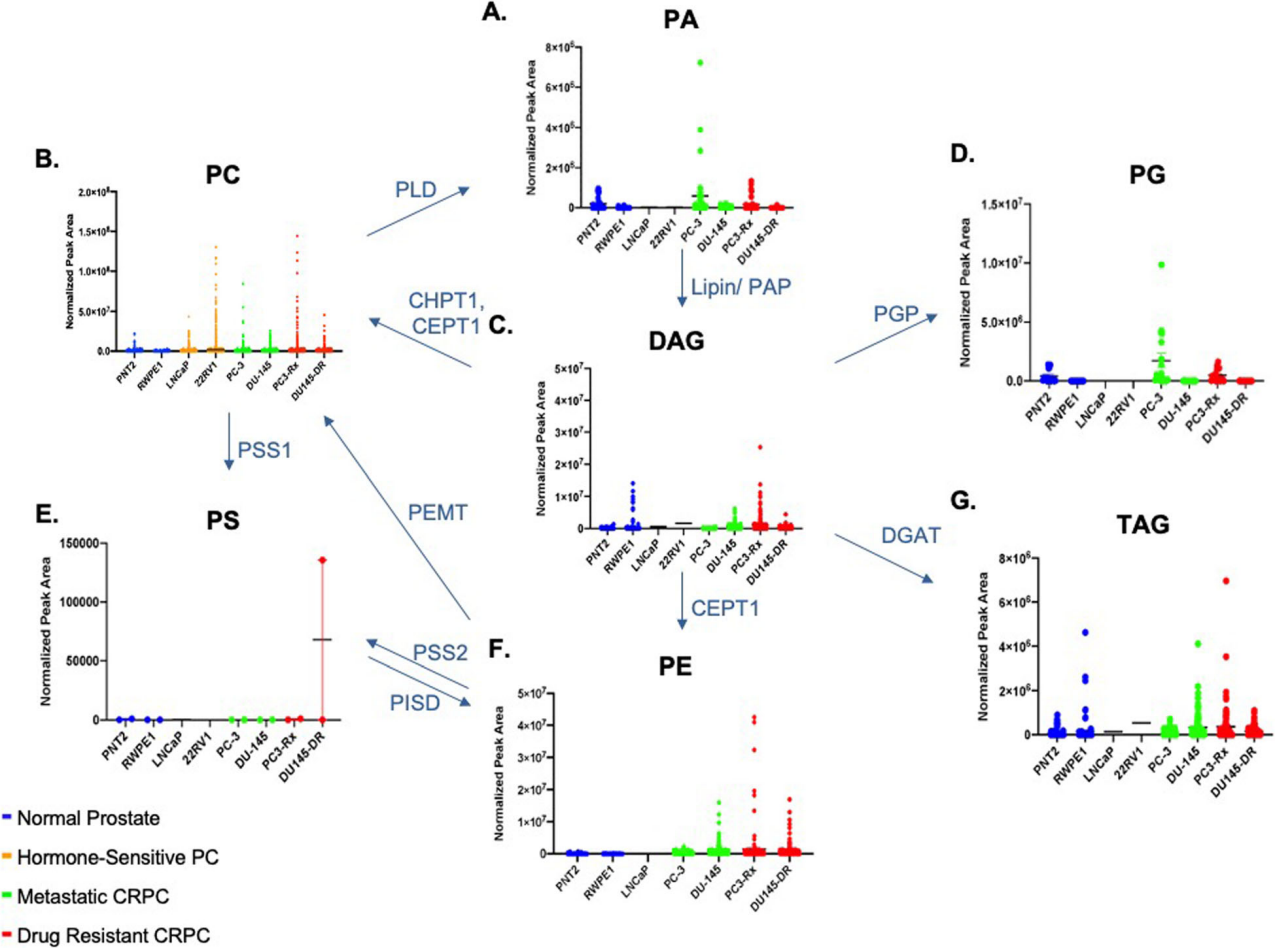
Alterations of phospholipid and acyl glycerol classes were observed in non-cancerous prostate cells (blue), hormone-sensitive (dark red), castration-resistant (CR) (green), and Docetaxel resistant (DR) (red) prostate cancer cells. LC-ESI-MS/MS analysis of these classes in comparison to the Kennedy Pathway demonstrates alterations of the lipid abundance within progressing prostate cancer cells. The enzymes mediating the remodeling are shown in blue along the arrows. CHPT1 Cholinephosphotransferase 1, CEPT1 Choline/ethanolaminephosphotransferase 1, DAG diacylglycerol, DGAT diacylglycerol acyltransferase, PA phosphatidic acid, PAP phosphatidic acid phosphatase, PC phosphatidylcholine, PLD Phospholipase D, PE phosphatidylethanolamine, PEMT Phosphatidylethanolamine-N-methyltransferase, PG phosphatidylglycerol, PGP phosphatidylglycerol phosphatase, PS phosphatidylserine, PSS1/ PSS2 phosphatidylserine synthase, PISD Phosphatidylserine decarboxylases, TAG triacylglycerol

**Fig. 4:**
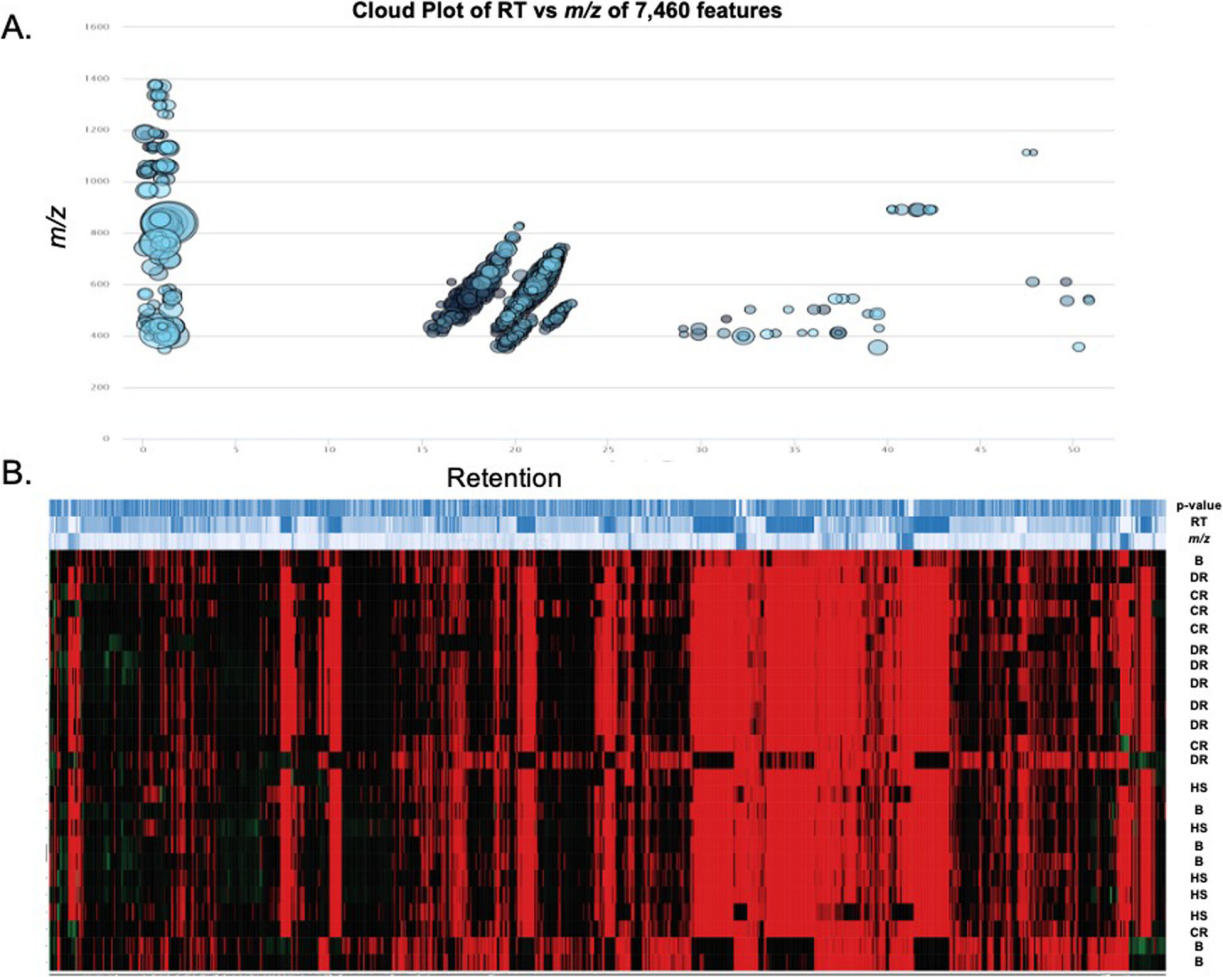
**a** Differential cloud plot demonstrating dysregulated features between hormone-sensitive, castration-resistant, Docetaxel resistant cells and non-cancerous cells (PNT2 and RWPE1) as determined by LC-ESI-MS/MS (*P*-value < 0.05 threshold, fold change > 1.5 threshold). **b** Differential expression of lipid features in non-cancerous prostate cells (**b**) as compared to hormone-sensitive (HS), castration-resistant (CR) and Docetaxel resistant (DR) prostate cancer cells. Only those features whose levels vary significantly (*P* < 0.05) are projected on the heat map. Each row represents a metabolite feature and each column represents a sample

Some studies have shown an alteration in lipid diversity in the blood and serum. Unfortunately, the consequence of this change in lipid diversity still remains unknown, but the current consensus is that changes in lipid composition is associated with altered behavior of cancer cells. Extensive studies have provided strong evidence for reprogramming of lipid metabolism in cancer through the Kennedy Pathway [4–6] (Supplemental Figure 2). To validate these data, and to further analyze lipid changes, an additional LC-ESI-MS/MS analysis was conducted comparing changes in lipid levels between hormone-sensitive, castration-resistant and drug resistant human prostate cancer cell lines in comparison to non-cancer prostate cell lines (Table 1). LC-ESI-MS/MS of phospholipid and acyl glycerol classes demonstrated alterations of abundance in prostate non-cancerous, hormone-sensitive, and metastatic CRPC cells (Fig. 3). This resulted in the identification of 7460 dysregulated ion features, encompassing 21 different lipid species (Fig. 4a). This was supported by a heat map analysis (Fig. 4b). Furthermore, OPLS-DA comparing hormone-sensitive cell lines to control cells in the positive mode (ESI+) showed distinct separation of each prostate cell line (Supplemental Figure 3A). This is further analyzed in (Supplemental Figure 3B), which presents a cloud plot demonstrating directional fold changes, significance, retention times and *m/z* values. This analysis identified 84 altered lipidomic features between hormone-sensitive cells as compared to normal cells. These lipids were also identified via heat map analysis (Supplemental Figure 3C). OPLS-DA showed clear separation between castration-resistant and non-cancerous prostate cancer cell lines (Supplemental Figure 4A), suggesting differential lipidomic profiles with the cell types. This was supported by a cloud plot analysis (Supplemental Figure 4B) that identified 45 lipids whose levels differed between castration-resistant cell-lines and non-cancer control cells. The relative abundance of each lipid species levels varied significantly across all samples identified in CRPC cell lines in comparison to normal cells (Supplemental Figure 4C).

**Table 1:**
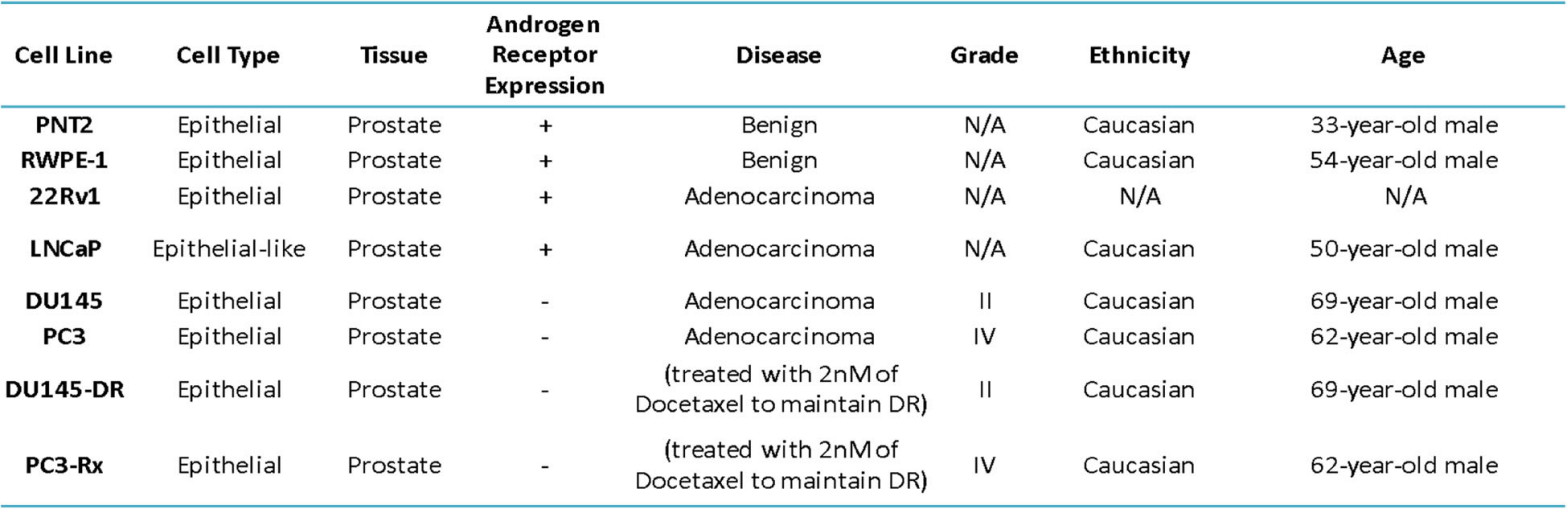
Prostate Cell Line Characteristics

**Table 2:** Prostate Cancer Population Studies from cBioPortal

Prostate Study	Number of Samples
Prostate Cancer (DKFZ, Cancer Cell 2018)	324
Prostate Cancer (MSK, 2019)	18
Metastatic Prostate Adenocarcinoma (MCTP, Nature 2012)	61
Metastatic Prostate Adenocarcinoma (SU2C/PCF Dream Team, PNAS 2019)	444
Metastatic Prostate Cancer (SU2C/PCF Dream Team, Cell 2015)	150
Metastatic castration-sensitive prostate cancer (MSK, Clin Cancer Res 2020)	424
Neuroendocrine Prostate Cancer (Multi-Institute, Nat Med 2016)	114
Prostate Adenocarcinoma (Broad/Cornell, Cell 2013)	57
Prostate Adenocarcinoma (CPC-GENE, Nature 2017)	477
Prostate Adenocarcinoma (MSKCC, 2020)	1465
Prostate Adenocarcinoma (MSKCC, Cancer Cell 2010)	240
Prostate Adenocarcinoma (SMMU, Eur Urol 2017)	65
Prostate Adenocarcinoma (TCGA, Cell 2015)	333
Prostate Adenocarcinoma Organoids (MSKCC, Cancer Cell 2014)	12
Prostate Cancer (MSKCC, JCO Precis Oncol 2017)	504
The Metastatic Prostate Cancer Project (Provisional, November 2019)	75

### Alterations in PC lipids

**Fig. 5:**
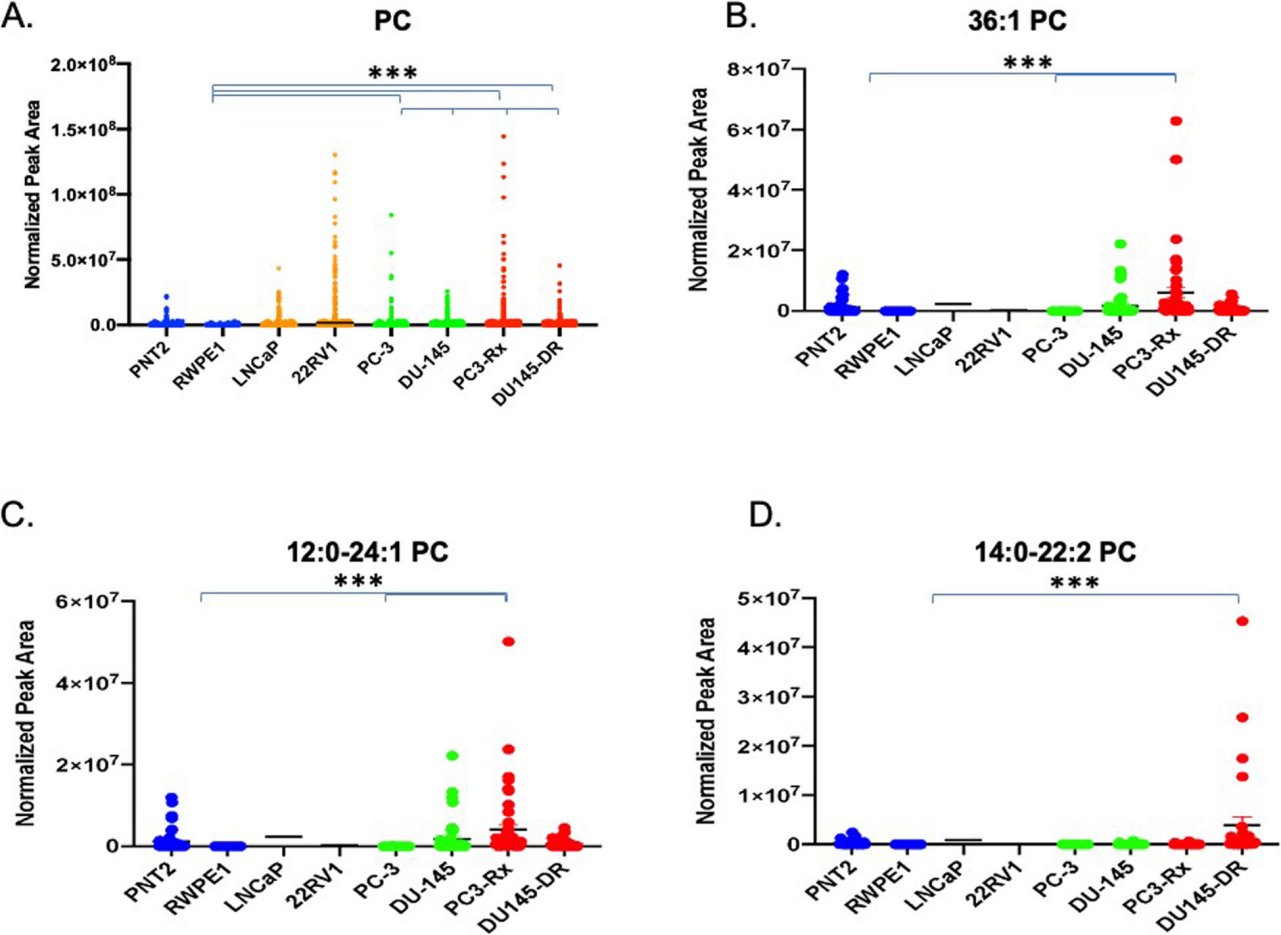
Comparison of phosphatidylcholine (PC) in non-cancerous (PNT2 and RWPE1) and hormone-sensitive (LNCaP and 22RV1), castration-resistant (PC-3 and DU-145) and Docetaxel resistant (PC3-Rx and DU145-DR) prostate cell lines. Data are indicative of 6 samples (6 distinct passages) per group and are expressed as mean ± the SEM (*q < 0.05 **q < 0.01*** q < 0.001). Each symbol represents an individual lipid feature as identified by MS/MS. Normalized peak areas between all cells are shown for **a** phosphatidylcholine (PC), **b** 36:1 PC **c** 12:0–24:1 PC and **d** 14:0-22:2 PC

**Fig. 6:**
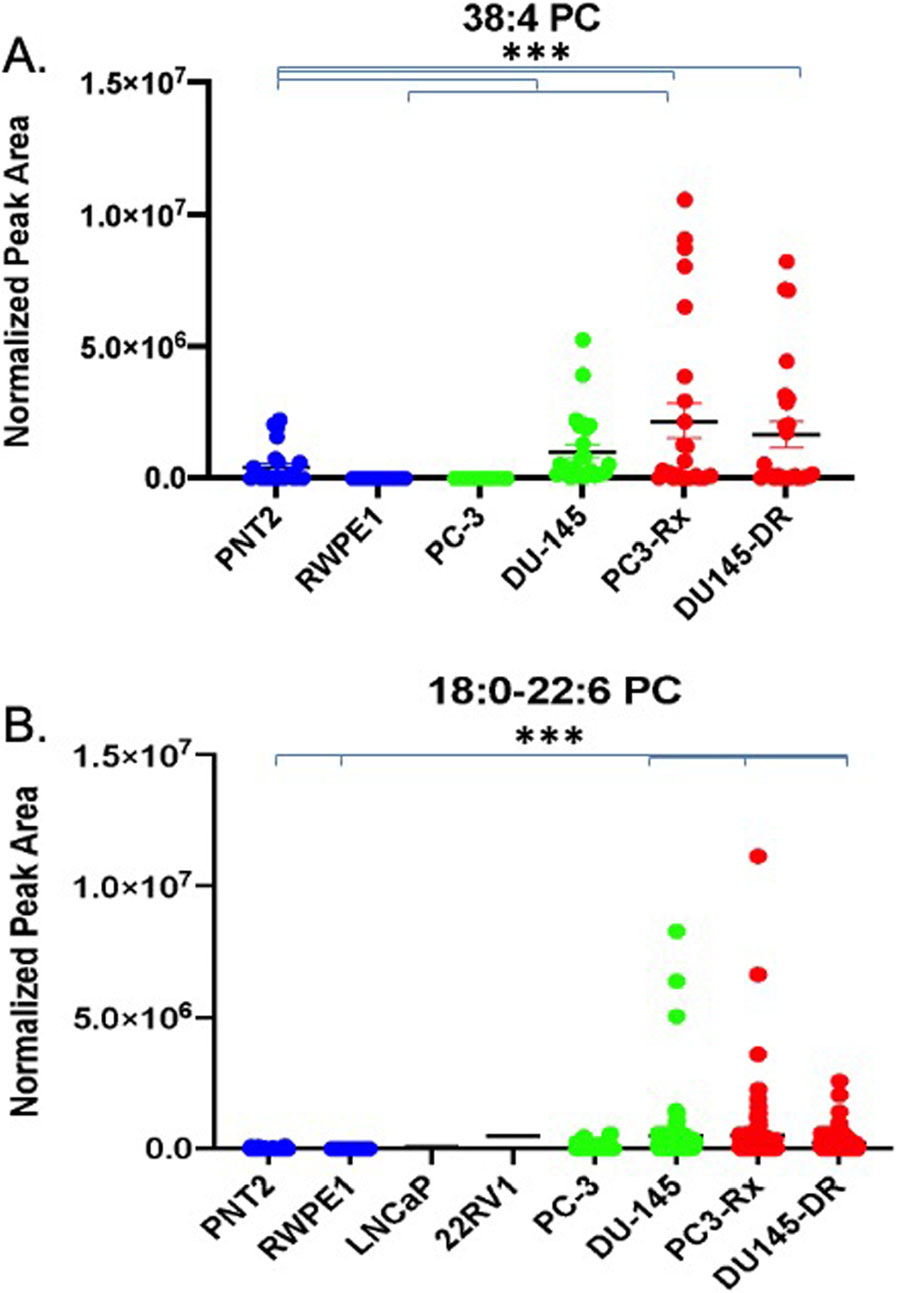
Comparison of **a** 38:4 and **b** 18:0–22:6 PC levels in non-cancerous (PNT2 and RWPE1), hormone-sensitive (LNCaP and 22RV1), castration-resistant (PC-3 and DU-145) and Docetaxel resistant (PC3-Rx and DU145-DR) prostate cell lines. Data are indicative of 6 samples (6 distinct passages) per group and are expressed as mean ± the SEM (*q < 0.05 **q < 0.01*** q < 0.001). Each symbol represents an individual lipid feature as identified by MS/MS

**Fig. 7:**
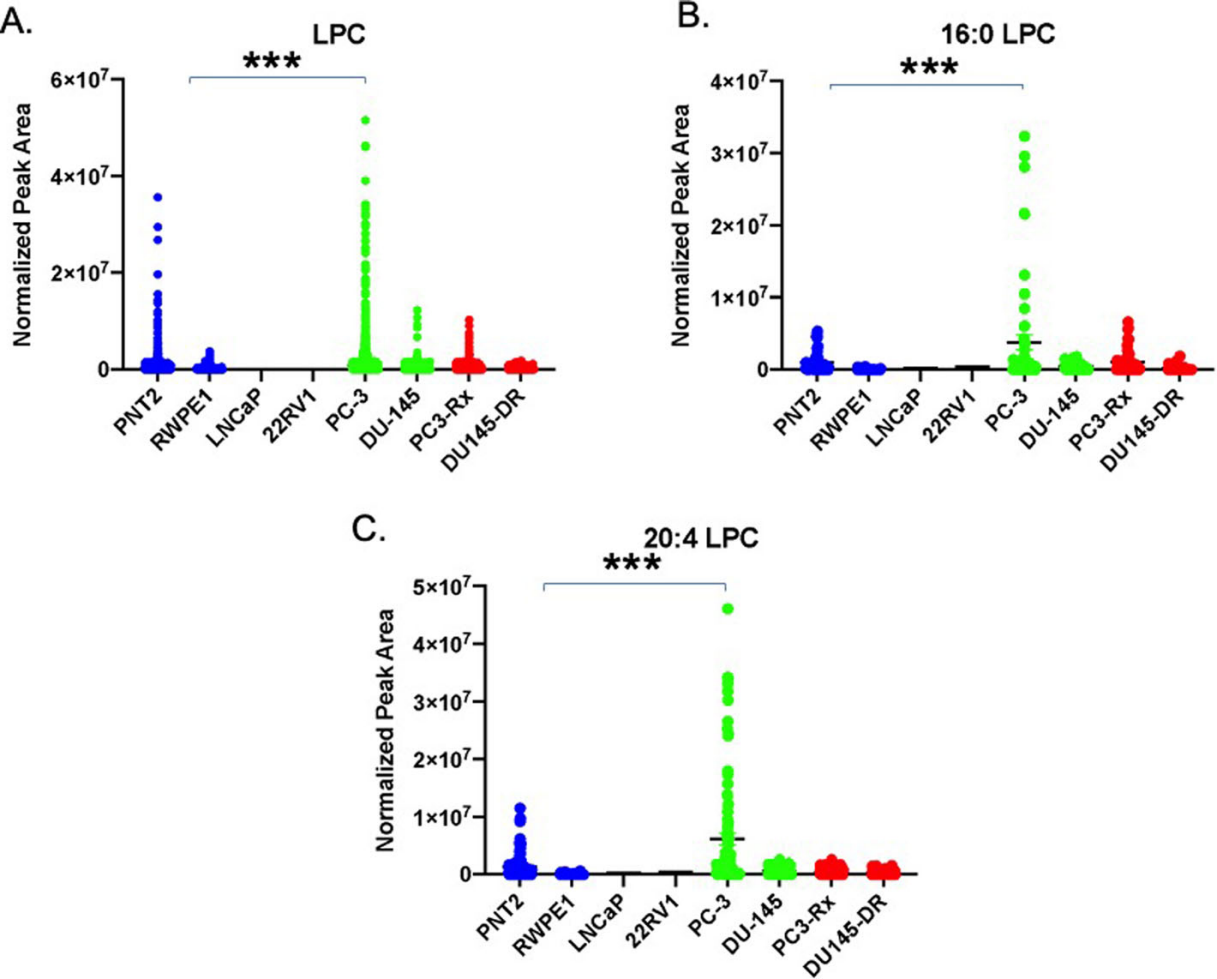
Comparison of **a** lysophosphocholine (LPC), **b** LPC and **c** 20:4 LPC levels in non-cancerous (PNT2 and RWPE1), hormone-sensitive (LNCaP and 22RV1), castration-resistant (PC-3 and DU-145) and Docetaxel resistant (PC3-Rx and DU145-DR) prostate cell lines. Data are indicative of 6 samples (6 distinct passages) per group and are expressed as mean ± the SEM (*q < 0.05 **q < 0.01*** q < 0.001). Each symbol represents an individual lipid feature as identified by MS/MS

In agreement with the LIPEA analysis, PC lipids were augmented in all six PCa cell lines analyzed, when compared to non-cancerous RWPE1 and PNT2 cells (Fig. 5a). Amongst the PC lipids, 36:1 PC was significantly increased in the PC3-Rx Docetaxel resistant cell type analyzed (Fig. 5b). Both 12:0–24:1 PC and 14:0–22:2 PC were also identified as a dominant PC species (Figs. 5c and d). Interestingly, 38:4 PC (Fig. 6a) was significantly enriched in both Docetaxel resistant cell types, as compared to the content level in both PC-3 and DU-145 parent and non-cancer control cells. 18:0–22:6 PC (Fig. 6b) was another dominant PC species that was increased in select cells. Most interestingly, LPC were enriched specifically in PC-3 parent cells, with 16:0 and 20:4 LPC being particularly prominent in this cell line (Fig. 7). These data support the LIPEA results, which suggested increased glycerophospholipid metabolism in prostate cancer cells.

**Fig. 8:**
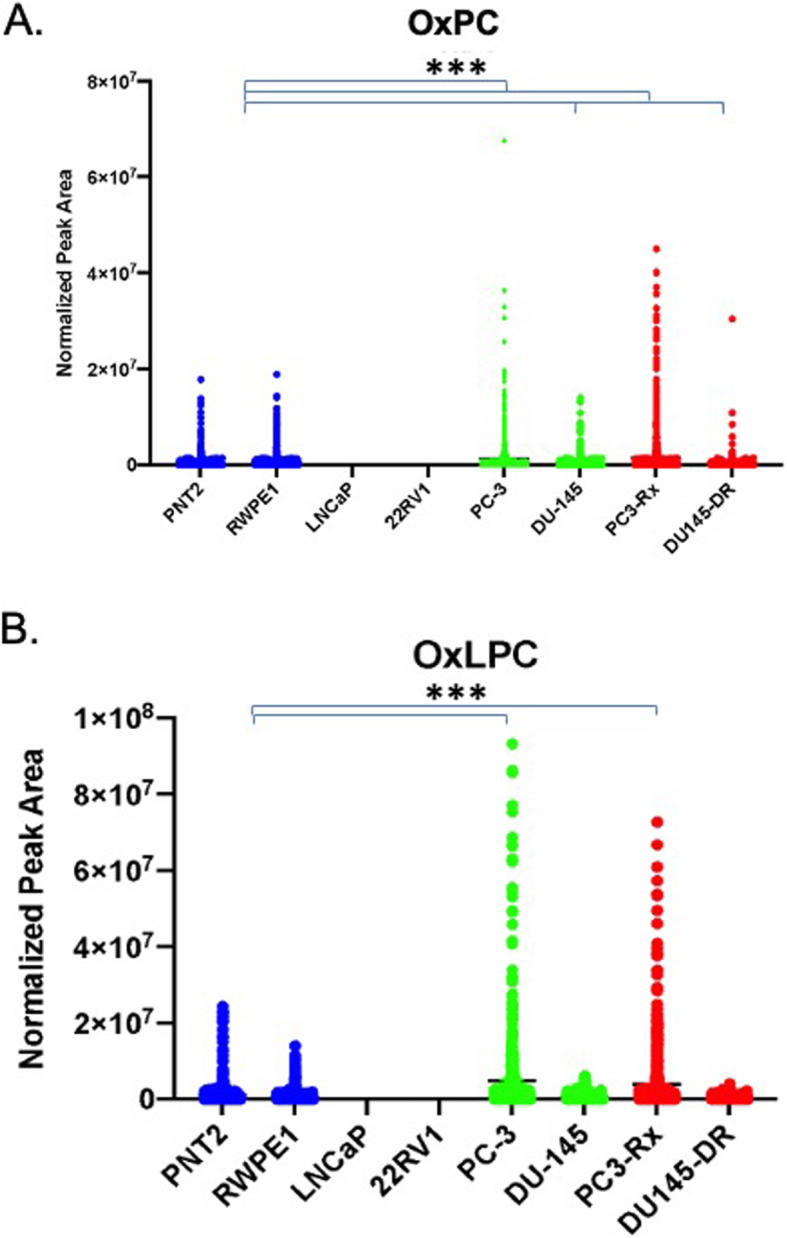
Comparison of oxidized phosphatidylcholines (OxPC) levels in non-cancerous (PNT2 and RWPE1), hormone-sensitive (LNCaP and 22RV1), castration-resistant (PC-3 and DU-145) and Docetaxel resistant (PC3-Rx and DU145-DR) prostate cell lines and media. Data are indicative of 6 samples (6 distinct passages) per group and are expressed as mean ± the SEM (*q < 0.05 **q < 0.01*** q < 0.001). Each symbol represents an individual lipid feature as identified by MS/MS. Normalized peak areas between all cells are shown for **a** oxidized phosphatidylcholine (OxPC), **b** oxidized lysophosphatidylcholine (OxLPC)

LIPEA also suggested increased pathways correlating to ferroptosis. This may correlate to an increase in oxidative stress and metabolism, among other events, in these cells. In support of this hypothesis, the levels of oxidized PC (OxPC) were enriched in prostate cancer cells as opposed to non-cancer cells (Fig. 8a). While a specific lipid species was not identified, the data did demonstrate an increase in oxidized LPC (OxLPC) in some cancer cells, especially PC-3 cells from both the parent and Docetaxel resistant strain. This correlates very well with data suggesting that LPC-species are specifically increased in PC-3 cells (Fig. 7).

### Alteration in PE lipids

PCs are not the only glycerophospholipids. As such, changes in PE, PG, PI and PS lipids were investigated as well. Overall, there was not any observable significant enrichments of Phosphatidylserine PS or Phosphatidylinositol (PI) lipid species between non-cancer and cancerous prostate cancer cells, with the exception of increased PA and PG in PC-3 cells when compared to PNT2 and RWPE1 cells (Supplemental Figure 5A and 5B). This trend was only observed when comparing androgen-insensitive cells to non-cancer cells and was not observed between parent PC-3 and DU-145 cells and their Docetaxel resistant counterparts (data not shown). A general enrichment of plasmalogens was also observed in most prostate cancer cells when compared to non-cancer cells (Supplemental Figure 5C); however, no observable trend was identified between the cancer cell lines.

**Fig. 9:**
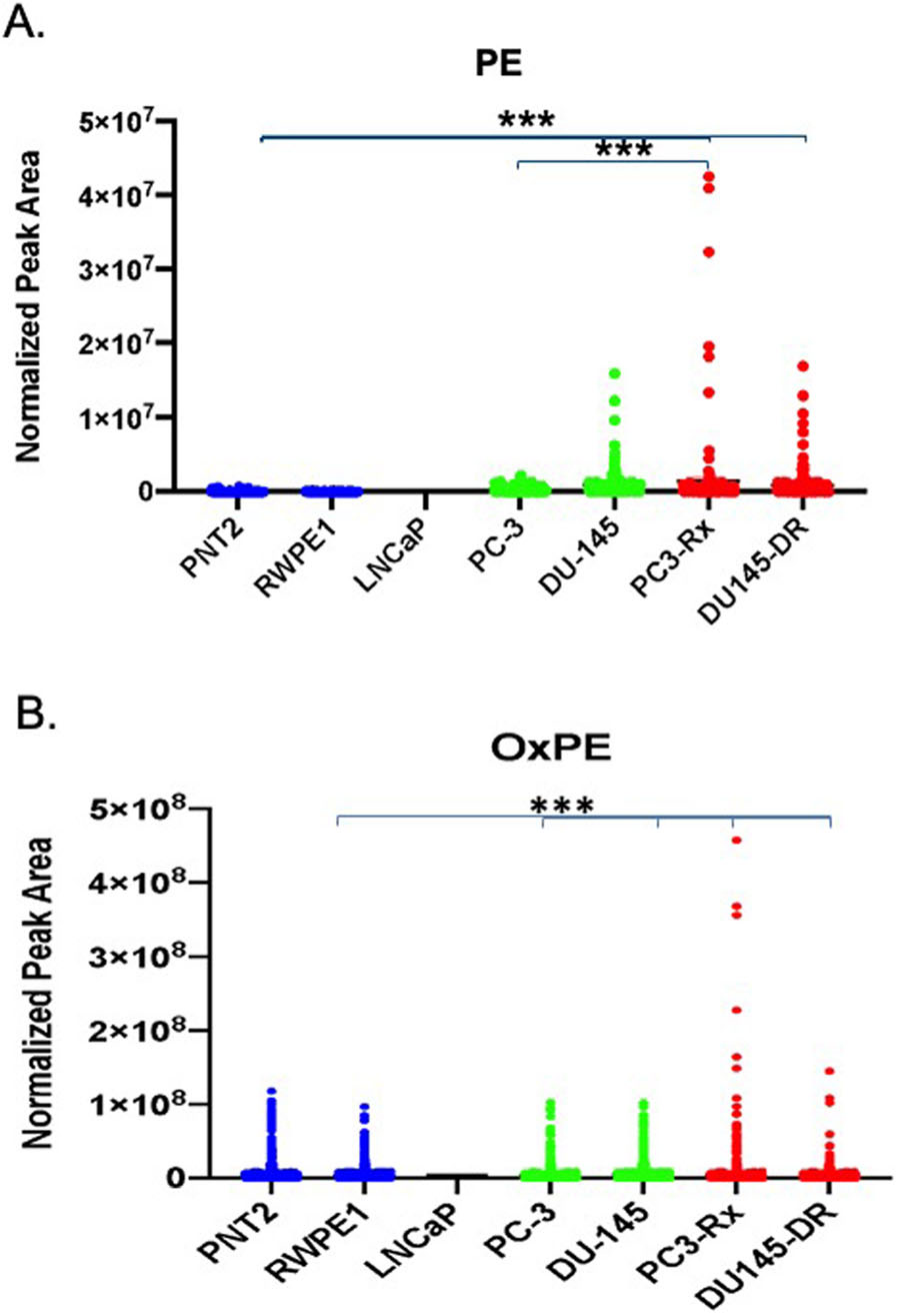
Comparison of phosphatidylethanolamine (PE) levels in non-cancerous (PNT2 and RWPE1), hormone-sensitive (LNCaP and 22RV1), castration-resistant (PC-3 and DU-145) and Docetaxel resistant (PC3-Rx and DU145-DR) prostate cell lines and media. Data are indicative of 6 samples (6 distinct passages) per group and are expressed as mean ± the SEM (*q < 0.05 **q < 0.01*** q < 0.001). Each symbol represents an individual lipid feature as identified by MS/MS. Normalized peak areas between all cells are shown for phosphatidylethanolamine (PE), **b** OxPE

In contrast to other glycerophospholipids, PE was significantly enriched in 22RV1, DU-145 and PC3-Rx cells as compared to non-cancer cells (Fig. 9a). Further, significant enrichment of PE species was observed in PC3-Rx cells as compared to its parent cell lines, or even to all other cells. 38:4 PE identified as 18:0–20:4 PE, was the most dominant of the PE lipid species in PC3-Rx cells, as compared to PC-3 parent control (Figs. 9b and c). Similar to PC lipids, OxPE was also significantly enriched in PC-3 and PC3-Rx cells, as compared to non-cancerous cells (Fig. 9b). A similar trend was seen for LPE, which similar to LPC, was enriched in PC-3 and PC3-Rx cells (Supplemental Figure 6).

### Alterations in sphingolipids

**Fig. 10:**
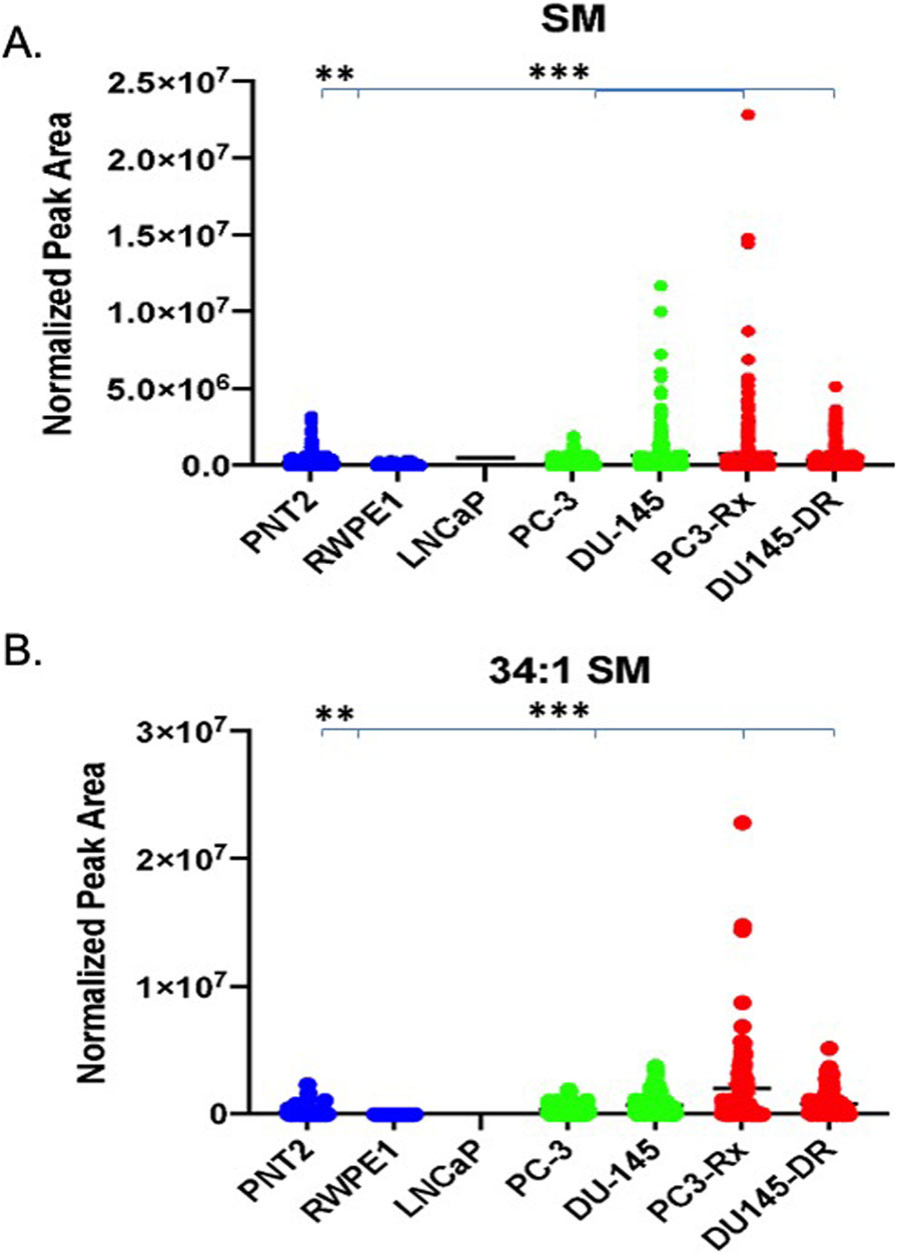
Comparison of sphingomyelin (SM) levels in non-cancerous (PNT2 and RWPE1), hormone-sensitive (LNCaP and 22RV1), castration-resistant (PC-3 and DU-145) and Docetaxel resistant (PC3-Rx and DU145-DR) prostate cell lines and media. Data are indicative of 6 samples (6 distinct passages) per group and are expressed as mean ± the SEM (*q < 0.05 **q < 0.01*** q < 0.001). Each symbol represents an individual lipid feature as identified by MS/MS. Normalized peak areas between all cells are shown for **a** sphingomyelin (SM) and **b** 34:1 + H SM

LIPEA also suggested an increase in sphingolipids metabolism and within the sphingolipid signaling pathways. In support of this, SM was significantly enriched in PC3-Rx cells as compared to PC-3 parent control and non-cancerous cells (Fig. 10a). 34:1 + H was the dominant SM species identified in PC3-Rx cells (Fig. 10b). Nonetheless, these data support LIPEA suggesting that sphingolipid metabolism and signaling are enriched in prostate cancer cells.

### Alterations in LPIN genes

**Fig. 11:**
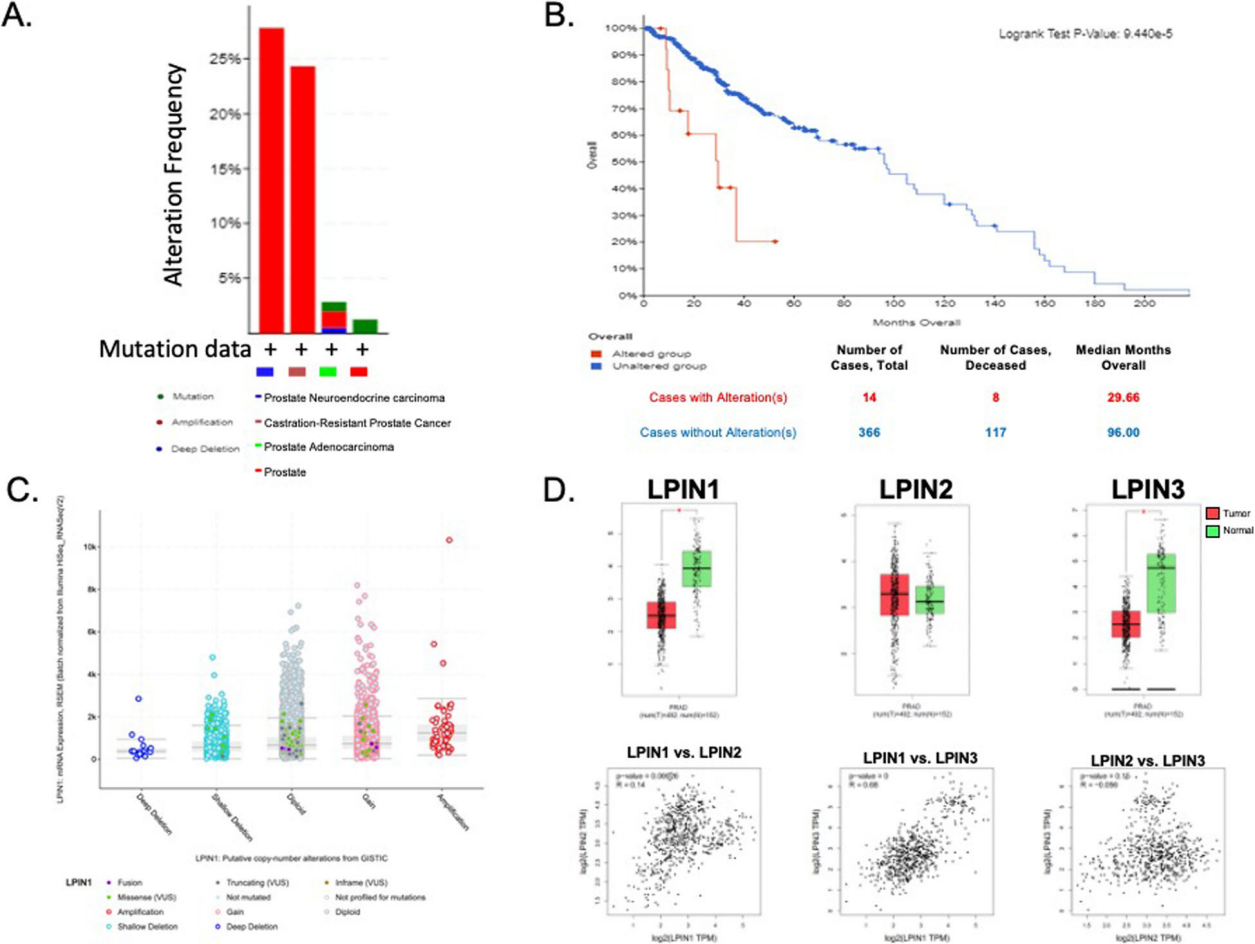
**a** Alterations in lipin genes LPIN1, LPIN2 and LPIN3 within four types of prostate cancer from sixteen studies. Amplification is the major alteration in all types, excluding general prostate cancer. **b** Overall survivability in prostate cancer patients with (red) or without (blue) lipin gene alteration. There are 28 samples with alterations in LPIN1, 37 samples from 30 patients with alterations in LPIN2 and 27 samples out of 26 patients with LPIN3 alterations. **c** mRNA expression**,** accurate transcript quantification from RNA-Seq data (RSEM) as a function of putative copy-number alterations in mutation type. There are 10,712 samples from 32 studies on the horizontal axis, 6961 samples from 16 studies on the vertical axis and 6909 samples from 16 studies at the intersection of the two axes. The figures were generated from cBioPortal for Cancer Genomics. **d** Variation in expression levels of lipin genes were observed in patients with (red) and without (green) prostate cancer. The method for differential analysis was a one-way ANOVA, using disease state (Tumor or Normal) as variable for calculating differential expression. Pair-wise gene expression correlations, using the Pearson method, of the lipin genes were analyzed based on TCGA and GTEx databases. The non-log scale for calculation and use the log-scale axis for visualization. The figures were generated from GEPIA 2. PRAD prostate adenocarcinoma, T tumor, N normal, TPM transcripts per million

Based on the LIPEA analysis performed in this study, glycerophospholipid metabolism was ranked the highest abnormal pathway and significantly associated with the set of lipids identified (Fig. 2). We further analyzed the alterations of phospholipid abundance and acyl glycerol classes between the PCa and non-cancer cell lines by studying lipin, one of the first enzymes involved in the Kennedy Pathway. There are three lipin proteins responsible for the regulation of PA to diacylglycerol (DAG). This conversion is a divergent point for the synthesis of triacylglycerol (TAG) and other phospholipids (PL). cBioPortal and GEPIA 2 were used to assess the possible contribution of lipins to prostate cancer. From the studies selected from cBioPortal, alterations in the LPIN1, LPIN2 and LPIN3 genes within prostate cancer, prostate adenocarcinoma, castration-resistant prostate cancer and prostate neuroendocrine carcinoma resulted in amplification being the major form of alteration (Fig. 11a). Overall survivability of patients within these studies differed with and without lipin gene alteration. For those with alterations, the median months survived after diagnosis was 29.7, whereas this was elongated to 96 months for those without alterations in these genes (Fig. 11b). mRNA expression for LPIN1 also showcases an overall higher amount of amplification occurring (Fig. 11c). Results from GEPIA 2 show expression levels of lipin genes varying between patients with and without prostate cancer, with higher significance in LPIN1 and LPIN3 (Fig. 11d). Additionally, gene expression between the three lipin genes demonstrate a strong correlation between LPIN1 and LPIN3 (Fig. 11d).

### Alterations in Lipin protein expression

**Fig. 12:**
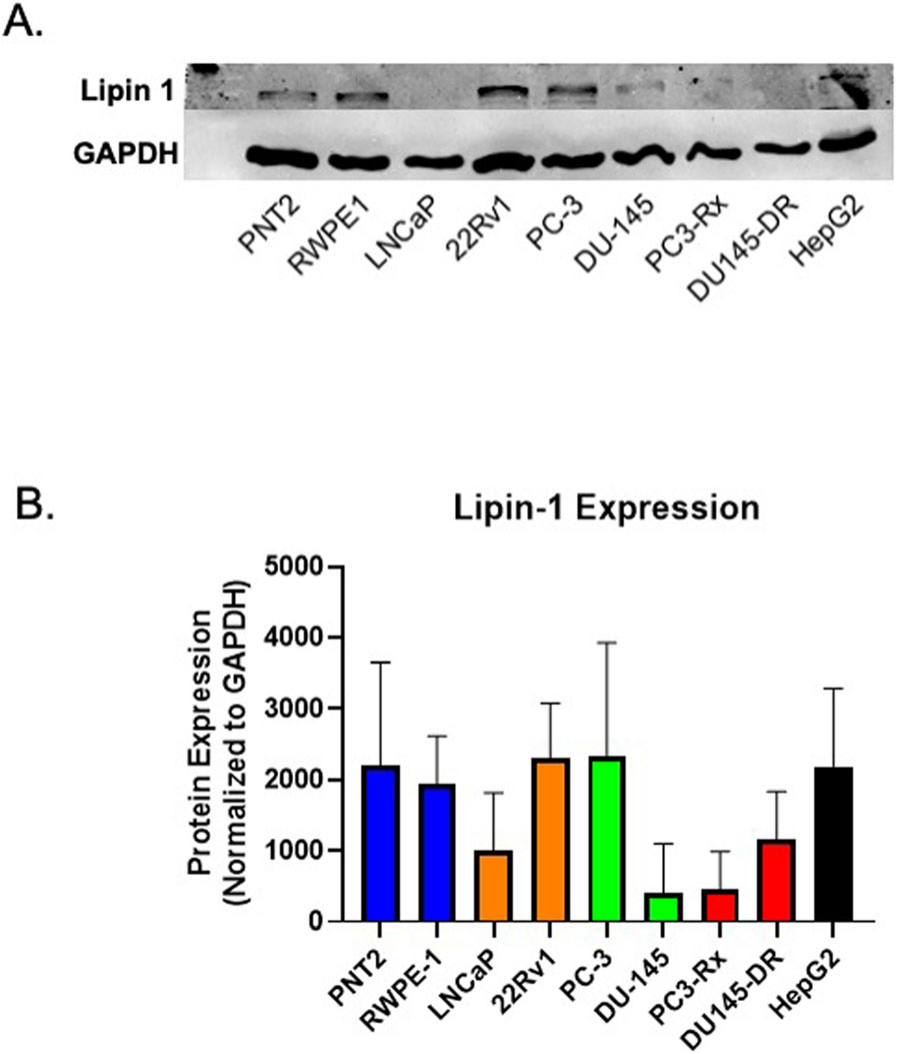
**a** Western blot for Lipin-1 (~ 125 kDa) and GAPDH expression (~ 36 kDa) in non-cancerous prostate cells (PNT2 and RWPE-1), hormone-sensitive (LNCaP and 22RV1), castration-resistant (CR) (PC-3 and DU-145), Docetaxel resistant (DR) (PC3-RX and DU145-DR) prostate cells and hepatocellular carcinoma (HepG2) cells (positive control). Immunoblots are representative of at least 3 (*n* = 3) separate experiments using 3 separate passages. **b** Densitometry of Lipin-1 protein normalized to GAPDH within non-cancerous prostate cells (blue), hormone-sensitive (orange), castration-resistant (CR) (green), Docetaxel resistant (DR) (red) prostate, and hepatocellular carcinoma (black) cell lines

To directly correlate the lipid alterations with the LIPEA pathway results, western blots with the lipin proteins were conducted. Lipin has a molecular weight of about 99 kDa but predicted bands around 125 kDa. With lipin-1 antibody, strong bands were noted at the predicted 125 kDa (Fig. 12b). The expression of lipin-1 in most cell lines correlated to the level PA, excluding the Docetaxel resistant ones (Figs. 3 and Fig. 12a). The decrease in lipin-1 expression in both PC3-Rx and DU145-DR is particularly perplexing as there appears to be an increase in DAG in PC3-Rx cell line, compared to PA (Fig. 3). Antibodies for lipin-2 and lipin-3 were tested alongside lipin-1, but these antibodies were unable to detect lipin-2 or lipin-3, despite the use of multiple antibodies. Further studies will be conducted to determine the other enzymatic effects of the Kennedy pathway.

## Discussion

Drug resistance in prostate cancer remains an unsolved challenge and is one of the primary drivers of low survivability among prostate cancer patients. While studies have identified lipid species as biomarkers in various cancers, fewer attempts have been made for drug resistance prostate cancer, due to the high complexity and diversity among lipid molecules. Recent evidence suggests elevated plasma levels of phospholipids are associated with an increased risk of PCa [33]. Unfortunately, these studies did not link individual lipids to the generation of drug resistance. The initial MS Peak to Pathway-Metaboanalyst approach identified multiple pathways altered in the panel of prostate cancer cells analyzed. Hierarchical clustering found several nodes that correlated to glycerophospholipid and sphingolipid metabolism. This is not surprising as the extraction methods used may be biased in this analysis towards glycerophospholipids. Further, Metaboanalyst is not perfected for lipids species. The later limitation was addressed by using the LIPEA pathway analysis, which was recently identified to mine pathways significantly associated with select lipid species. LIPEA, which works with compound IDs for lipids found in KEGG, also identified glycerophospholipid metabolism as being significantly enriched in PCa cells compared to non-cancer cells.

The enrichment of glycerophospholipid metabolism aligned with the fact that lipids generated from glycerophospholipid metabolism are also increased in PCa cells, including LPC and LPE. Metabolites from these molecules regulate many signaling pathways, as well as cell growth.

LIPEA also identified sphingolipid metabolism as being enriched in PCa cells, which agrees with the analysis generated by MetaboAnalyst and hierarchical clustering. Several studies have shown that sphingolipid-mediated gene expression plays a critical role in cancer by several mechanisms [34–36]. This includes the regulation of lipid-lipid interaction, membrane structure and/or regulation of the interaction of membrane proteins with the membrane bilayer [37].

The identification of ferroptosis as being enriched in PCa cells was unexpected. Ferroptosis is a regulated form of iron-dependent, non-apoptotic cell death [38]. Ferroptosis can be driven by extensive lipid peroxidation that alters the physical properties of the membrane or degrades reactive compounds that cross-link DNA or proteins [39–41]. Ferroptosis is linked to numerous diseases of the kidney, heart, liver and brain [38, 42]. To date, only a few studies have linked changes in ferroptosis specifically to prostate cancer. Mechanistically, blocking enzymes such as DECR1, PANX2 and GPX4 can inhibit tumor growth with universal implications in understanding both resistance and metabolic events [42, 43]. Excitingly, one recent study did identify that certain cancer cells were vulnerable to ferroptic cell death induced by inhibition of a lipid peroxidase pathway as a feature of therapy-resistant cancer cells [42–46]. As such, these data support this study by demonstrating that an enrichment of lipids corresponding to ferroptosis aligns with drug-resistance PCa cells. Further, these data suggest that targeting ferroptosis may alter the sensitivity of PCa cells to Docetaxel.

Data demonstrating the significant enrichment of three lipid classes (namely, PC, PE, and SM) in hormone-sensitive cells in comparison to normal control cells is consistent with previous reports in plasma from PCa patients [33, 43, 44]. Elevated concentrations of SM in plasma were previously reported in patients with PCa in comparison to the control group [45]. These data further support the previously stated hypothesis that sphingolipids have a potential role in regulating PCa cells response to chemotherapy [46]. Further, these data support that these lipids may serve to identify drug-resistance prostate cancer. Finally, these data show that at least part of the lipidomic profile seen in PCa patients can be recapitulated in vitro (see below). This allows for a model to begin to investigate the molecular mechanism involved in changes in lipids during prostate cancer progression and the development of drug resistance.

Data from this study also showed elevated levels of plasmalogens in PCa cells as compared to non-cancerous cells. An increase in plasmalogens in these cell lines has not been previously reported. However, a previous study reported increased levels of plasmalogen phospholipids in neoplastic human breast tissue as compared to benign tissue and correlated this with metastatic properties of human cancer [47]. The significance of these data to prostate cancer progression or drug resistance is not known.

The increase in specific PC lipids, such as 36:1 and 12:0–24:1 in PC-3 cells, but not in DU-145 cells mirrored data seen for LPC, OxPC and OxLPC. Elevated PC in plasma has been associated with prostate cancer progression. For example, 20:4 LPC was suggested as a top biomarker for prostate cancer [48]. LPC has also been shown to be elevated in tissues exposed to radio/chemotherapy treatments [49–51]; however, it is not known if chemotherapy increased LPC levels, or if LPC was elevated prior to treatment [52]. Other reports have demonstrated that the LPC is increased in ovarian cancer patients and the fatty acid composition of this LPC is changed [53].

The elevation of PE in prostate cancer cells agrees with the findings of increased PE in patient plasma samples [43, 54], as well as in prostate cancer cells (LNCaP, 22RV-1 and DU-145), as compared to PNT2 cells [44]. PE has also been detected in high abundance in exosomes derived from PC-3 cells [55]. Similar to LPC levels in PC-3 cells, enrichment in LPE was primarily seen in PC-3 cells from both parent and Docetaxel resistant cell lines. Previous studies also compared PE plasmalogens in normal, benign and neoplastic samples from human prostate, breast, and lung tissues, and suggested that these are lipid tumor markers for distinguishing between benign vs. neoplastic tissue and identifying in-vivo metastatic progression.

While this study represents one of the most comprehensive analyses of lipid composition in prostate cells to date, it is limited by the fact that the actual concentrations for lipid species were not provided. This was in part intentional and these data are meant to springboard further studies focusing on how specific lipids identified are being altered in these cells. Further, it is important to note that standards for many of the specific lipid species, as opposed to general classes, are not commercially available. Future studies will focus on quantifying these specific lipids in these cells as well as validating their existence in human plasma.

These data, as well as previous studies demonstrate that there are fundamental differences between the lipidome of cancer and non-cancer cells [44, 56–58]. Non-cancer cells typically exhibit neutral total membrane charge due to the presence of zwitterionic phospholipids (PC and SM) on the outer leaflet of the membrane and PS and PE located in the inner leaflet of the membrane [59–61]. Unlike normal cells, cancer cells typically lose their capacity to maintain asymmetrical distribution leading to abnormal exposure of (PS and PE) to the cell outer membrane and/or PC and SM to cytosolic leaflet causing changes in cell signaling and downstream gene expression. Although lipids are not encoded by the genome, targeting lipid enzymes is one way to control lipid homeostasis. For example, lipins catalyze the dephosphorylation of phosphatidic acid to diacylglycerol, which is a precursor of triacylglycerol and phospholipids [62, 63]. As such, lipins also function as co-transcriptional regulators of lipid homeostasis. Lipin-1 protein expression was detected in multiple cancer cell lines in this study, agreeing with at least one other study that showed lipin-1 expression in select cells [64]. Previous studies also demonstrated that the Lipin-1 knockdown repressed proliferation of prostate and breast cancer cells [65]. However, there is still a gap in knowledge as to how these processes shape the progression of drug-resistant cancer or mediate lipid levels in prostate cancer cells. Our data demonstrates a correlation between the amplification of lipins in prostate cancer patients and decreased survivability. Our data further show that decreased expression of lipins correlate to prostate cancer. Data in cells suggest a link between drug resistance and lipin-1 levels as well as to differences in the levels of PA and DAG, at least in one cell line.

Lipin-1 is a key enzyme in the Kennedy Pathway, which is a primary pathway for glycerophospholipid synthesis. The alterations in lipin-1, -2 and -3 expression in prostate cancer patients, and correlation between lipin-1 expression and PA and DAG in some select cell lines, suggest that these enzymes may regulate lipid levels in prostate cancer. Further, lipin alterations may indicate aggressiveness and drug resistance.

### Study strengths and limitations

The overall key findings from this study are the: (1) identification of a unique lipid signature for drug resistant prostate cancer, (2) determination of aberrant lipid pathways in drug resistant CRPC progression and (3) the correlation in lipin expression in prostate cancer patient and cells to aggressiveness and survivability. These findings are strengthened by the use of multiple cell lines, multiple analysis (Shotgun and Targeted Lipidomics), a pathway analysis and subsequent analysis correlated to patient samples that identified a potential lipid metabolism enzyme. The limitation of this study is that there is no consensus on proper data processing protocols. For lipidomics to be implemented in a clinical setting, one must account for factors perturbing lipid measurements. With these are numerous sample preparation protocols, and many factors that reduce the accuracy and precision of lipid measurements that are not fully understood. Thus, the data in cells are limited by the fact their relevance to clinical data is not known. Nevertheless, these data lay the groundwork for future studies assessing changes in the specific lipids and proteins identified in this study. The data are also limited in that the lipid levels are semi-quantitative. Lipid concentrations measured across labs are often drastically different. Furthermore, quantification is problematic due to unavailable lipid standards to cover the diverse species within a given lipidome. Therefore, strategies to select the best internal standards for the pertinent lipids identified in this study are needed. A final limitation is that we were unable to detect lipin-2 and -3, despite the use of multiple antibodies. Further, lipin activity was not accessed. As such, a complete understanding of the role of lipin in mediating the PA/DAG axis in prostate cancer is not possible.

## Conclusion

Global lipid pathway analysis suggested glycerophospholipid metabolism is the bottleneck contributor to the tumorigenic lipids that drive drug resistant prostate cancer progression, with an integrated lipidomic/transcriptomic high gene signature score correlated to poor survival. These data further identify the novel finding that the lipidomic profile of drug-resistant prostate cancer cells also differs, even from their parent cells. Thus, the use of lipid profiling in cell culture is critical in assessing the function of various lipid species.

An abundance of lipid species can be collected via non-invasive procedures and easily monitored using human biological fluids, which include blood and urine. As such, these lipids may be useful for identifying drug-resistant prostate cancer in vivo. These findings are important as understanding lipidomics, including the underlying molecular machinery of lipid metabolism, would assist in the discovery of novel and potential targets and develop new predictors for personalized cancer treatments. Finally, these data support the conclusion that changes in these lipidomic profiles mirror those reported in patient samples.

## Supplementary Information

**Additional file 1: Figure S1**

**Additional file 2: Figure S2**

**Additional file 3: Figure S3**

**Additional file 4: Figure S4**

**Additional file 5: Figure S5**

**Additional file 6: Figure S6**

**Additional file 7:.** Supplementary Data

**Additional file 8:.** Supplemental Document

## Data Availability

According to the policy of BMC, we would like to inform that all the datasets analyzed during the current study are available from the corresponding author upon reasonable request. Authors will make data available on National Metabolomics Data Repository (NMDR).

## Abbreviations

LIPEA: Lipid Pathway Enrichment Analysis
CRPC/CR: Castration-resistant prostate cancer
DU145-DR/ DR: Docetaxel resistant DU-145 cells
PC3-Rx/ DR: Docetaxel resistant PC-3 cells
LC-ESI-MS/MS: Liquid Chromatography- Electrospray ionization- tandem/ mass spectrometry
LPC: Lysophosphatidylcholine
OxLPC: Oxidized lysophosphatidylcholine
OxPC: Oxidized phosphatidylcholine
OxPE: Oxidized phosphatidylethanolamine
PA: Phosphatidic acid
PC: Phosphatidylcholine
PE: Phosphatidylethanolamine
PG: Phosphatidylglycerol
PS: Phosphatidylserine
PL: Phospholipids
PCa: prostate cancer
SM: Sphingomyelin
CHPT1: Cholinephosphotransferase 1
CEPT1: Choline/ethanolaminephosphotransferase 1
DAG: Diacylglycerol
DGAT: Diacylglycerol acyltransferase
PAP: Phosphatidic acid phosphatase
PLD: Phospholipase D
PEMT: Phosphatidylethanolamine-N-methyltransferase
PGP: Phosphatidylglycerol phosphatase
PSS1/ PSS2: phosphatidylserine synthase
PISD: Phosphatidylserine decarboxylases,
TAG: Triacylglycerol

## References

[1] Chandrasekar T (2015). Mechanisms of resistance in castration-resistant prostate cancer (CRPC). Transl Androl Urol.

[2] George DJ, Kantoff PW, Lin DW (2011). New and emerging treatments for advanced prostate cancer. Clin Adv Hematol Oncol.

[3] Hultsch S (2018). Association of tamoxifen resistance and lipid reprogramming in breast cancer. BMC Cancer.

[4] Cheng C (2015). Glucose-mediated N-glycosylation of SCAP is essential for SREBP-1 activation and tumor growth. Cancer Cell.

[5] Guo, D., et al., *EGFR signaling through an Akt-SREBP-1–dependent, rapamycin-resistant pathway sensitizes glioblastomas to antilipogenic therapy.* Sci. Signal., 2009. 2(101): p. ra82-ra82. 10.1126/scisignal.2000446 PMC2978002 20009104

[6] Guo D (2011). An LXR agonist promotes glioblastoma cell death through inhibition of an EGFR/AKT/SREBP-1/LDLR–dependent pathway. Cancer discovery.

[7] Acevedo, A., et al., *LIPEA: Lipid Pathway Enrichment Analysis.* bioRxiv, 2018: p. 274969.

[8] Twum-Ampofo J (2016). Metabolic targets for potential prostate cancer therapeutics. Curr Opin Oncol.

[9] Ngo DC (2015). Introduction to the molecular basis of cancer metabolism and the Warburg effect. Mol Biol Rep.

[10] Medes G, Thomas A, Weinhouse S (1953). Metabolism of neoplastic tissue. IV. A study of lipid synthesis in neoplastic tissue slices in vitro. Cancer Res.

[11] Ookhtens M (1984). Liver and adipose tissue contributions to newly formed fatty acids in an ascites tumor. Am J Phys.

[12] Beloribi-Djefaflia S, Vasseur S, Guillaumond F (2016). Lipid metabolic reprogramming in cancer cells. Oncogenesis.

[13] DeBerardinis RJ, Chandel NS (2016). Fundamentals of cancer metabolism. Sci Adv.

[14] DeBerardinis RJ (2008). The biology of Cancer: metabolic reprogramming fuels cell growth and proliferation. Cell Metab.

[15] Bligh EG, Dyer WJ (1959). A rapid method of total lipid extraction and purification. Can J Biochem Physiol.

[16] Bartlett GR (1959). Phosphorus assay in column chromatography. J Biol Chem.

[17] Maes E (2015). Determination of variation parameters as a crucial step in designing TMT-based clinical proteomics experiments. PLoS One.

[18] Kinsey GR (2008). Decreased iPLA2γ expression induces lipid peroxidation and cell death and sensitizes cells to oxidant-induced apoptosis. J Lipid Res.

[19] Zhang L, Peterson BL, Cummings BS (2005). The effect of inhibition of Ca2+−independent phospholipase A2 on chemotherapeutic-induced death and phospholipid profiles in renal cells. Biochem Pharmacol.

[20] Peterson B (2008). Alterations in phospholipid and fatty acid lipid profiles in primary neocortical cells during oxidant-induced cell injury. Chem Biol Interact.

[21] Kalli A (2013). Evaluation and optimization of mass spectrometric settings during data-dependent acquisition mode: focus on LTQ-Orbitrap mass analyzers. J Proteome Res.

[22] Pluskal T (2010). MZmine 2: modular framework for processing, visualizing, and analyzing mass spectrometry-based molecular profile data. BMC Bioinformatics.

[23] Tautenhahn R (2012). XCMS online: a web-based platform to process untargeted metabolomic data. Anal Chem.

[24] Koelmel JP (2017). LipidMatch: an automated workflow for rule-based lipid identification using untargeted high-resolution tandem mass spectrometry data. BMC Bioinformatics.

[25] Koelmel JP (2017). Expanding Lipidome coverage using LC-MS/MS data-dependent acquisition with automated exclusion list generation. J Am Soc Mass Spectrom.

[26] Chong J (2018). MetaboAnalyst 4.0: towards more transparent and integrative metabolomics analysis. Nucleic Acids Res.

[27] Kinsey GR (2008). Decreased iPLA2gamma expression induces lipid peroxidation and cell death and sensitizes cells to oxidant-induced apoptosis. J Lipid Res.

[28] Cerami E (2012). The cBio cancer genomics portal: an open platform for exploring multidimensional cancer genomics data. Cancer Discov.

[29] Gao J (2013). Integrative analysis of complex cancer genomics and clinical profiles using the cBioPortal. Sci Signal.

[30] Tang Z (2019). GEPIA2: an enhanced web server for large-scale expression profiling and interactive analysis. Nucleic Acids Res.

[31] Trygg J, Wold S (2002). Orthogonal projections to latent structures (O-PLS). J Chemom.

[32] Trygg J, Holmes E, Lundstedt T (2007). Chemometrics in metabonomics. J Proteome Res.

[33] Richman EL (2012). Choline intake and risk of lethal prostate cancer: incidence and survival. Am J Clin Nutr.

[34] Fernandis AZ, Wenk MR (2007). Membrane lipids as signaling molecules. Curr Opin Lipidol.

[35] Brzozowski JS (2018). Lipidomic profiling of extracellular vesicles derived from prostate and prostate cancer cell lines. Lipids Health Dis.

[36] Ponnusamy S (2010). Sphingolipids and cancer: ceramide and sphingosine-1-phosphate in the regulation of cell death and drug resistance. Future Oncol.

[37] van Meer G, Voelker DR, Feigenson GW (2008). Membrane lipids: where they are and how they behave. Nat Rev Mol Cell Biol.

[38] Dixon SJ (2012). Ferroptosis: an iron-dependent form of nonapoptotic cell death. Cell.

[39] Latunde-Dada GO (2017). Ferroptosis: role of lipid peroxidation, iron and ferritinophagy. Biochim Biophys Acta Gen Subj.

[40] Agmon E (2018). Modeling the effects of lipid peroxidation during ferroptosis on membrane properties. Sci Rep.

[41] Agmon E, Stockwell BR (2017). Lipid homeostasis and regulated cell death. Curr Opin Chem Biol.

[42] Stockwell BR (2017). Ferroptosis: a regulated cell death Nexus linking metabolism, redox biology, and disease. Cell.

[43] Lin HM (2017). A distinct plasma lipid signature associated with poor prognosis in castration-resistant prostate cancer. Int J Cancer.

[44] Sorvina A (2018). Lipid profiles of prostate cancer cells. Oncotarget.

[45] Cheng JC (2013). Radiation-induced acid ceramidase confers prostate cancer resistance and tumor relapse. J Clin Invest.

[46] Ogretmen B, Hannun YA (2004). Biologically active sphingolipids in cancer pathogenesis and treatment. Nat Rev Cancer.

[47] Merchant TE (1991). Phospholipid profiles of human colon cancer using 31P magnetic resonance spectroscopy. Int J Color Dis.

[48] Perrotti F (2016). Advances in Lipidomics for Cancer biomarkers discovery. Int J Mol Sci.

[49] Perry DK, Hannun YA (1998). The role of ceramide in cell signaling. Biochim Biophys Acta.

[50] Taha TA, Mullen TD, Obeid LM (2006). A house divided: ceramide, sphingosine, and sphingosine-1-phosphate in programmed cell death. Biochim Biophys Acta.

[51] Wang X-Z (1999). Aberrant sphingolipid signaling is involved in the resistance of prostate Cancer cell lines to chemotherapy. Cancer Res.

[52] Schneider G (2014). Bioactive lipids, LPC and LPA, are novel prometastatic factors and their tissue levels increase in response to radio/chemotherapy. Molecular cancer research : MCR.

[53] Okita M (1997). Elevated levels and altered fatty acid composition of plasma lysophosphatidylcholine (lysoPC) in ovarian cancer patients. Int J Cancer.

[54] Zhou X (2012). Identification of plasma lipid biomarkers for prostate cancer by lipidomics and bioinformatics. PLoS One.

[55] Phuyal S (2015). The ether lipid precursor hexadecylglycerol stimulates the release and changes the composition of exosomes derived from PC-3 cells. J Biol Chem.

[56] Lisec J (2019). Cancer cell lipid class homeostasis is altered under nutrient-deprivation but stable under hypoxia. BMC Cancer.

[57] Burch TC (2015). Comparative Metabolomic and Lipidomic analysis of phenotype stratified prostate cells. PLoS One.

[58] Jung JH (2015). Phospholipids of tumor extracellular vesicles stratify gefitinib-resistant nonsmall cell lung cancer cells from gefitinib-sensitive cells. Proteomics.

[59] Bevers EM, Comfurius P, Zwaal RF (1996). Regulatory mechanisms in maintenance and modulation of transmembrane lipid asymmetry: pathophysiological implications. Lupus.

[60] Escribá PV (2006). Membrane-lipid therapy: a new approach in molecular medicine. Trends Mol Med.

[61] Escriba PV (2008). Membranes: a meeting point for lipids, proteins and therapies. J Cell Mol Med.

[62] Zhang TY (2013). Management of castrate resistant prostate cancer-recent advances and optimal sequence of treatments. Curr Urol Rep.

[63] Pascual F, Carman GM (2013). Phosphatidate phosphatase, a key regulator of lipid homeostasis. Biochim Biophys Acta.

[64] Liu Y (2006). Fatty acid oxidation is a dominant bioenergetic pathway in prostate cancer. Prostate Cancer Prostatic Dis.

[65] Brohée L (2015). Lipin-1 regulates cancer cell phenotype and is a potential target to potentiate rapamycin treatment. Oncotarget.

