# Supplementary figures and images for: Identification of lipidomic profiles associated with drug-resistant prostate cancer cells
Source: Lipids Health Dis. 2021 Feb 17;20:15. doi: 10.1186/s12944-021-01437-5 (PMC7890620; doi:10.1186/s12944-021-01437-5)

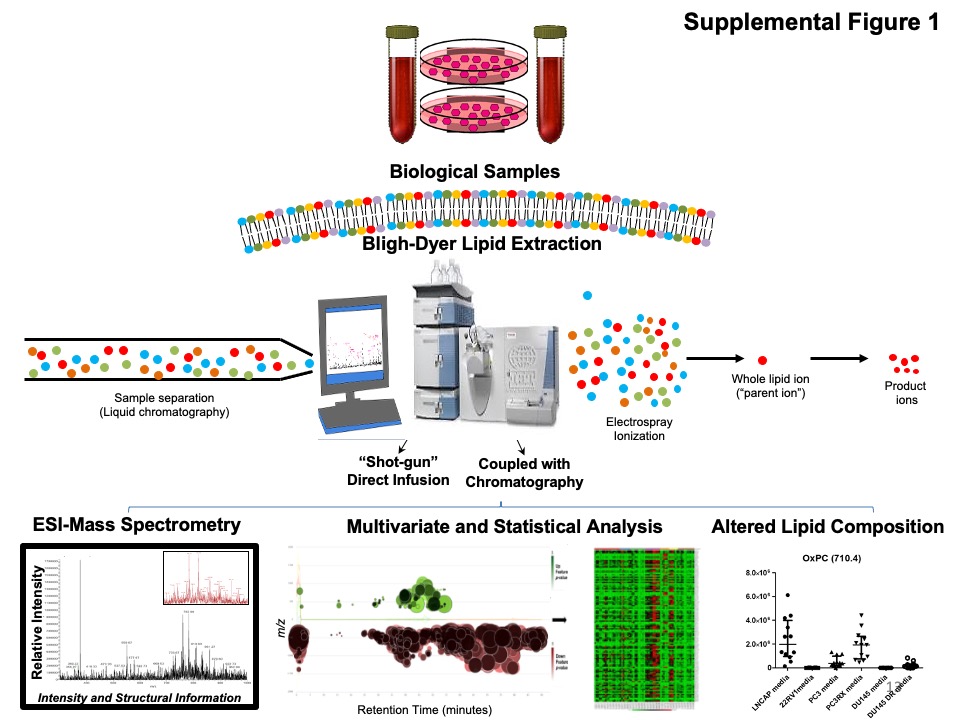

Supplement: Supplementary file 1 — Additional file 1: Figure S1 [file 12944_2021_1437_MOESM1_ESM.jpeg]

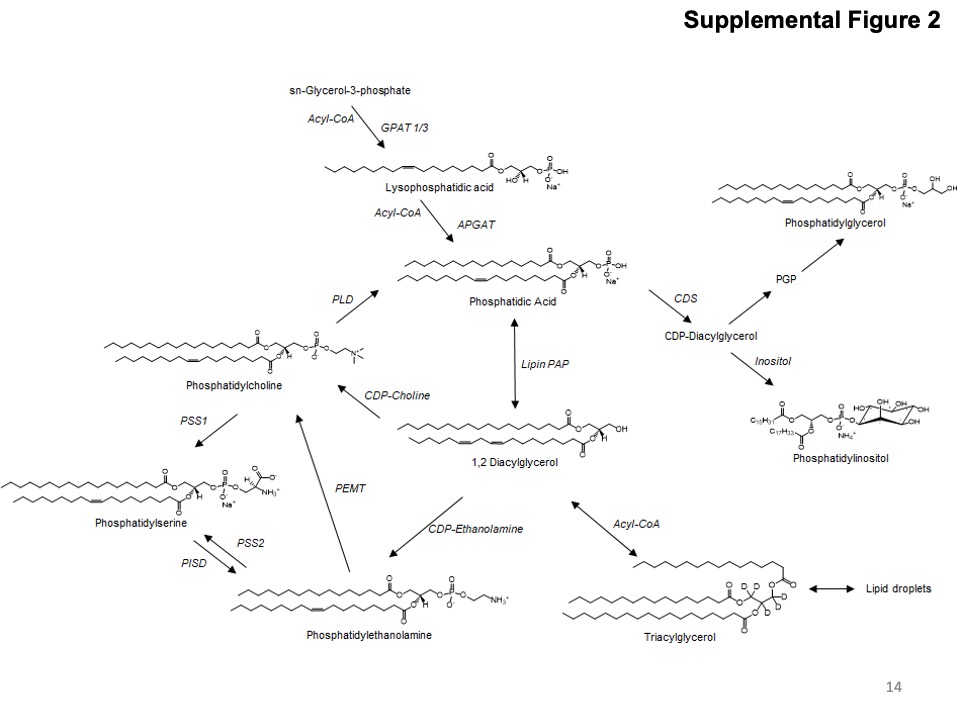

Supplement: Supplementary file 2 — Additional file 2: Figure S2 [file 12944_2021_1437_MOESM2_ESM.jpeg]

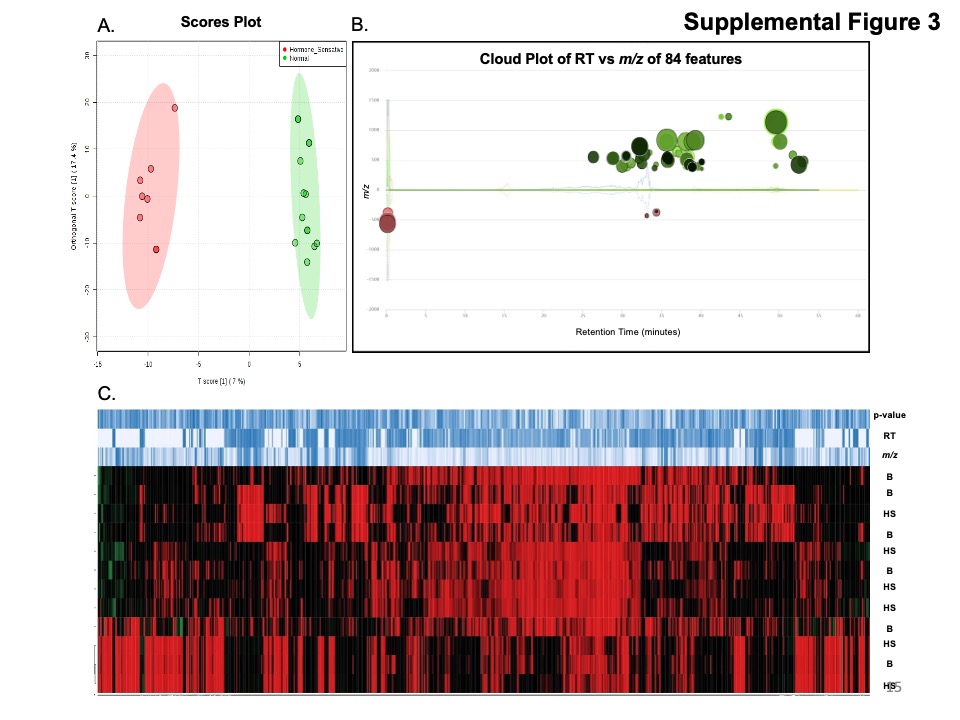

Supplement: Supplementary file 3 — Additional file 3: Figure S3 [file 12944_2021_1437_MOESM3_ESM.jpeg]

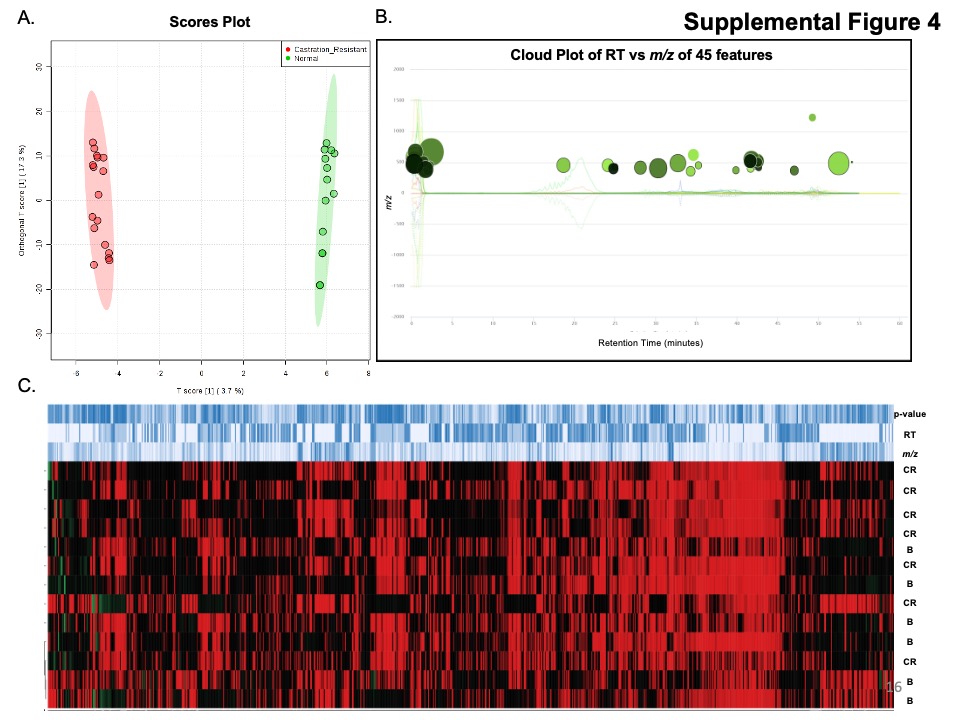

Supplement: Supplementary file 4 — Additional file 4: Figure S4 [file 12944_2021_1437_MOESM4_ESM.jpeg]

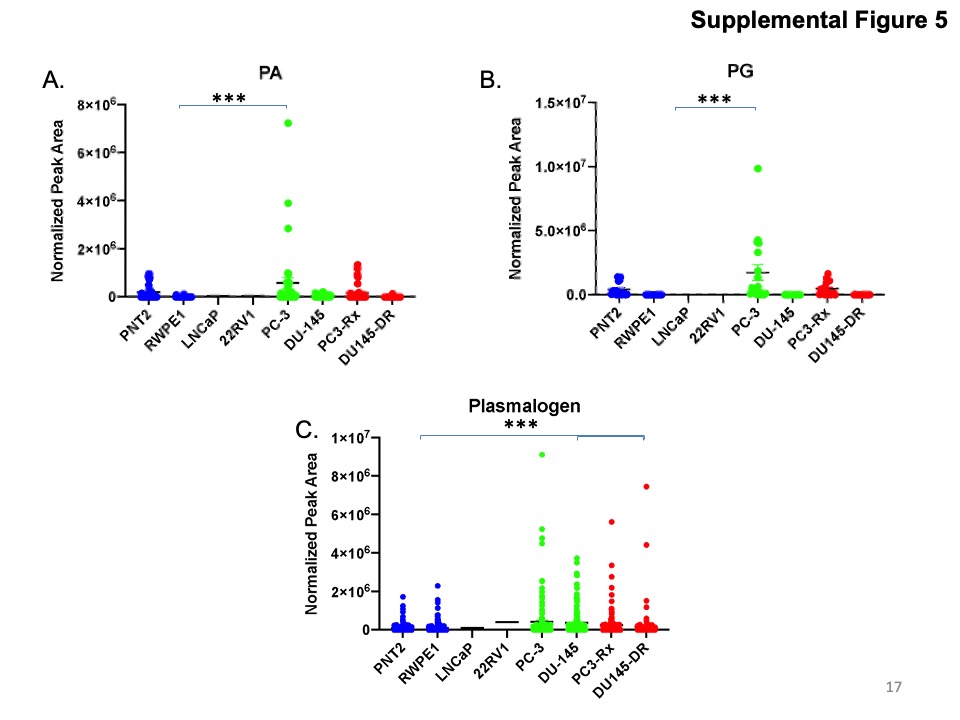

Supplement: Supplementary file 5 — Additional file 5: Figure S5 [file 12944_2021_1437_MOESM5_ESM.jpeg]

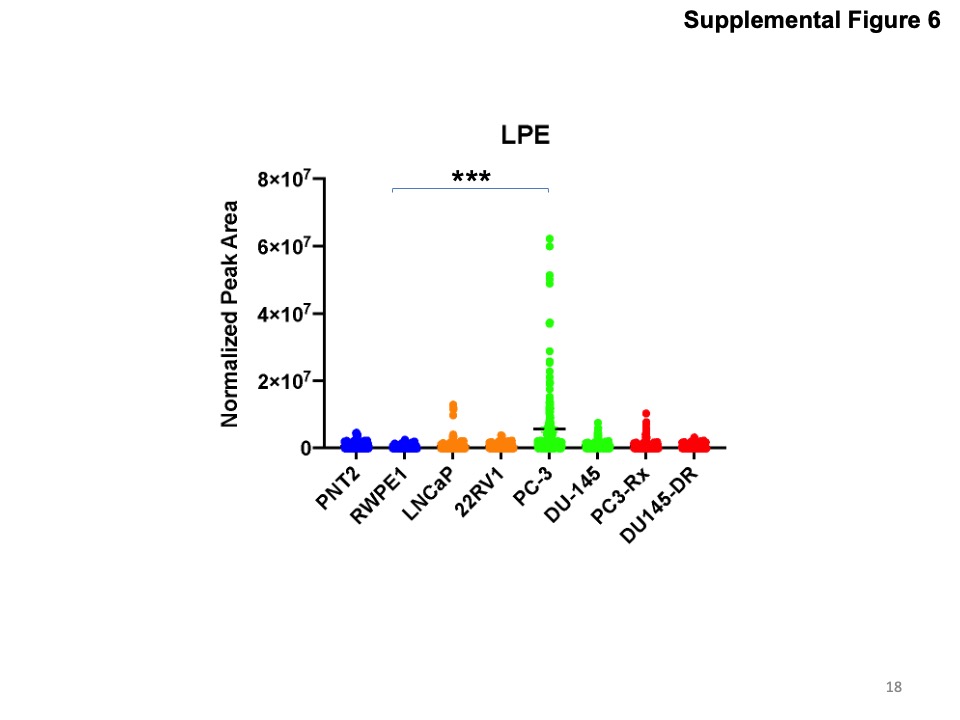

Supplement: Supplementary file 6 — Additional file 6: Figure S6 [file 12944_2021_1437_MOESM6_ESM.jpeg]
