# Supplementary material for: Identification of lipidomic profiles associated with drug-resistant prostate cancer cells
Source: Lipids Health Dis. 2021 Feb 17;20:15. doi: 10.1186/s12944-021-01437-5 (PMC7890620; doi:10.1186/s12944-021-01437-5)
Supplement: Supplementary file 7 — Additional file 7:. Supplementary Data [file 12944_2021_1437_MOESM7_ESM.docx]

**Supplemental Data**

**Identification of Lipidomic Profiles Associated with Drug-Resistant Prostate Cancer Cells**

Lishann M. Ingram^1^, Morgan C. Finnerty^1*^, Maryam Mansoura^1*^, Chau-Wen Chou^2^, Brian S. Cummings^1,3^

*Co-second authors

Affiliations:

^1^ Pharmaceutical and Biomedical Sciences, University of Georgia, Athens, GA, USA

^2^ Proteomics and Mass Spectrometry Facility (PAMS), Department of Chemistry, University of Georgia, Athens, GA, USA

^3^ Interdisciplinary Toxicology Program, University of Georgia, Athens, GA, USA

Corresponding Author:

Brian S. Cummings, Ph.D.; 450 College of Pharmacy South, University of Georgia Athens, GA 30602, Phone: 706-542-3792 Fax: 706-542-5358 E-Mail: briansc@uga.edu

**Keywords:** Drug resistance, Lipids, Lipidomics, Lipid metabolism, Lipid species, Mass Spectrometry, Metastasis, Prostate Cancer, Prostate

**Supplemental Table 1: LIPEA Pathway Enrichment**

| Pathway name | Pathway lipids | Converted lipids (number) | Converted lipids (percentage) | Converted lipids (list) | *p*-value | Benjamini correction | Bonferroni correction |
| --- | --- | --- | --- | --- | --- | --- | --- |
| Glycerophospholipid metabolism | 26 | 8 | 53% | C02737, C04438, C00416, C04230, C00157, C05973, C04233, C00350 | 5.20E-08 | 1.66E-06 | 1.66E-06 |
| Sphingolipid metabolism | 21 | 3 | 20% | C00195, C00550, C12126 | 0.0145 | 0.0656 | 0.4069 |
| Ferroptosis | 11 | 3 | 20% | C21480, C21481, C21484 | 0.0021 | 0.0148 | 0.0591 |
| Sphingolipid signaling pathways | 9 | 3 | 20% | C00195, C12126, C00550 | 0.0011 | 0.0103 | 0.0310 |
| Necroptosis | 4 | 2 | 13% | C00195, C00550 | 0.0001 | 0.0020 | 0.0039 |
| Retrograde endocannabinoid signaling | 8 | 2 | 13% | C00157, C00350 | 0.0164 | 0.0656 | 0.4595 |
| Phospholipase D signaling pathways | 7 | 1 | 6% | C00416 | 0.0037 | 0.0209 | 0.1046 |
| alpha-Linolenic acid metabolism | 23 | 1 | 6% | C00157 | 0.8837 | 0.8837 | 1.0000 |
| Glycerolipid metabolism | 15 | 1 | 6% | C00416 | 0.4939 | 0.5122 | 1.0000 |
| Autophagy - other | 3 | 1 | 6% | C00350 | 0.4649 | 0.5007 | 1.0000 |
| Autophagy - animal | 4 | 1 | 6% | C00350 | 0.3328 | 0.3727 | 1.0000 |
| Linoleic acid metabolism | 25 | 1 | 6% | C00157 | 0.3328 | 0.3727 | 1.0000 |
| Arachidonic acid metabolism | 75 | 1 | 6% | C00157 | 0.2559 | 0.3116 | 1.0000 |
| Glycine, serine and threonine metabolism | 3 | 1 | 6% | C02737 | 0.1013 | 0.1575 | 1.0000 |
| Glycosylphosphatidylinositol (GPI)-anchor biosynthesis | 3 | 1 | 6% | C00350 | 0.0769 | 0.1435 | 1.0000 |
| Neurotrophin signaling pathways | 3 | 1 | 6% | C00195 | 0.1709 | 0.2278 | 1.0000 |
| cAMP signaling pathway | 5 | 1 | 6% | C00416 | 0.0769 | 0.1435 | 1.0000 |
| Fc gamma R-mediated phagocytosis | 6 | 1 | 6% | C00416 | 0.1251 | 0.1843 | 1.0000 |
| GnRH signaling pathway | 3 | 1 | 6% | C00416 | 0.0769 | 0.1435 | 1.0000 |
| Phosphatidylinositol signaling system | 11 | 1 | 6% | C00416 | 0.1483 | 0.2076 | 1.0000 |
| AGE-RAGE signaling pathway in diabetic complications | 2 | 1 | 6% | C00195 | 0.0769 | 0.1435 | 1.0000 |
| Insulin resistance | 4 | 1 | 6% | C00195 | 0.0769 | 0.1435 | 1.0000 |
| Fat digestion and absorption | 8 | 1 | 6% | C00416 | 0.1013 | 0.1575 | 1.0000 |
| Adipocytokine signaling pathway | 3 | 1 | 6% | C00195 | 0.1013 | 0.1575 | 1.0000 |

**Supplemental Table 2: Selected Prostate Studies from cBioPortal**

**
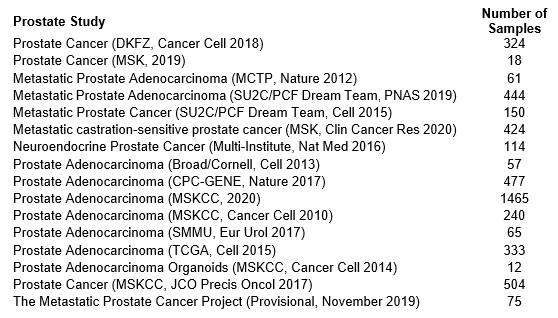
**

**Supplemental Table 3: Antibodies for Western Blots**


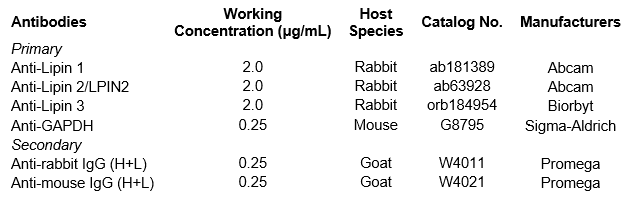


**Supplemental Figure 1.** **Lipidomic profiling and data processing approach**. This study utilized *in vitro* models that included non-cancerous, hormone sensitive, CRPC and drug-resistant human prostate cell lines combined with both an untargeted shotgun approach (ESI-MS) and quantitative HPLC-ESI-Orbitrap-MS lipidomic analysis. Lipid extracts were obtained by Bligh-Dyer (methanol/chloroform/water) extraction. Shotgun analysis was performed using commercially available standards. For target-based lipidomics, LC separation yielded specific lipid class separation prior to ESI/MS/MS enabling enhanced detection of lipids that are suppressed in the shotgun approach. Reverse-phase LC methods were examined for the class-specific separation of lipids species. Post data acquisition for both shotgun and HPLC-ESI/MS/MS lipidomics were based on multiple methods including isotope, carbon number (to IS) and ionization efficiency-based corrections. Online resources and software's utilized included LipidMatch, LipidNormalizer, Metaboanalyst, LIPID MAPS, XCMS, MZmine, SeeMS and TOPPview

**Supplemental Figure 2**. Lipid synthesis in prostate cancer cell progression. The enzymes mediating the remodeling are shown along the arrows. CHPT1 Cholinephosphotransferase 1, CEPT1 Choline/ethanolaminephosphotransferase 1, DAG diacylglycerol, DGAT diacylglycerol acyltransferase,PA phosphatidic acid, PAP phosphatidic acid phosphatase, PC phosphatidylcholine, PLD Phospholipase D, PE phosphatidylethanolamine, PEMT Phosphatidylethanolamine-N-methyltransferase, PG phosphatidylglycerol, PGP phosphatidylglycerol phosphatase, PS phosphatidylserine, PSS1/ PSS2 phosphatidylserine synthase, PISD Phosphatidylserine decarboxylases,

TAG triacylglycerol.

**Supplemental Figure 3**. **A**) Multiple variable analysis (MVA) of lipid features isolated from hormone sensitive LNCaP and 22RV1 cells. **B)** Differential cloud plot demonstrating dysregulated features between hormone-sensitive cells and non-cancerous cells PNT2 and RWPE1 (*p-value* <= 0.05 threshold, fold change >= 1.5 threshold). Up-regulated features (features that have a positive fold change) are graphed above the x-axis shown in green while down-regulated features (features that have a negative fold change) shown in red are graphed below the x-axis. **C**) Differential expression of lipid features in non-cancerous prostate cells (N) as compared to hormone-sensitive (HS) prostate cancer cells. Only those features who levels varied significantly (p < 0.05) are projected on the heat map. Rows represents a metabolite feature and each column represents a sample.

**Supplemental Figure 4**. **A**) Multiple variable analysis (MVA) of lipid features isolated from castration-resistant PC-3 and DU-145 cells. **B**) Differential cloud plot demonstrating dysregulated features between hormone-sensitive cells and non-cancerous cells (PNT2 and RWPE1) (*p*-value <= 0.05 threshold, fold change >= 1.5 threshold). Up-regulated features (features that have a positive fold change) are graphed above the x-axis shown in green while down-regulated features (features that have a negative fold change), shown in red, are graphed below the x-axis. **C**) Differential expression of lipid features in non-cancerous prostate cells (N) as compared to castration-resistant (CR) prostate cancer cells. Only those features who levels that vary significantly (p < 0.05) are projected on the heat map. Each row in **C**. represents a metabolite feature and each column represents a sample

**Supplemental Figure 5.** Comparison of additional glycerophospholipids in non-cancerous (PNT2 and RWPE1) and castration-resistant (PC-3 and DU-145) prostate cell lines. Data are indicative of 6 samples (6 distinct passages) per group and are expressed as mean ± the SEM (*q < 0.05 **q < 0.01*** q < 0.001). Each symbol represents an individual lipid feature as identified by MS/MS. Normalized peak areas between castration-resistant and control cells are shown for **A)** phosphatidic acid (PA), **B)** phosphatidylglycerol (PG) and **C)** plasmalogens.

**Supplemental Figure 6**. Comparison of lysophosphatidylethanolamine (LPE) levels in non-cancerous (PNT2 and RWPE1), hormone-sensitive (LNCaP and 22RV1), castration-resistant (PC-3 and DU-145) and Docetaxel resistant (PC3-Rx and DU145-DR) prostate cell lines and media. Data are indicative of 6 samples (6 distinct passages) per group and are expressed as mean ± SEM (*q < 0.05 **q < 0.01*** q < 0.001). Each symbol represents an individual lipid feature as identified by MS/MS.
