# Supplementary material for: Identification of lipidomic profiles associated with drug-resistant prostate cancer cells
Source: Lipids Health Dis. 2021 Feb 17;20:15. doi: 10.1186/s12944-021-01437-5 (PMC7890620; doi:10.1186/s12944-021-01437-5)
Supplement: Supplementary file 3 [file 12944_2021_1437_MOESM8_ESM.docx]

Identification of Lipidomic Profiles Associated...

By: Lishann Ingram

As of: Jan 5, 2021 8:07:20 AM
8,892 words - 60 matches - 3 sources

Similarity Index

10%

Mode: Similarity Report

paper text:

Identification of Lipidomic Profiles Associated with Drug-Resistant Prostate Cancer Cells Lishann M. Ingram1, Morgan Cora Finnerty1, Maryam Mansoura1, Chau-Wen Chou2, Brian S. Cummings1,3 Affiliations: 1Pharmaceutical and

Biomedical Sciences, University of Georgia, Athens, GA, USA 1

2Proteomics and Mass Spectrometry Facility (PAMS), Department of Chemistry,

University of Georgia, Athens, GA, USA 1

3Interdisciplinary

Toxicology Program, University of Georgia, Athens, GA, USA Corresponding Author: Brian S. Cummings, Ph.D.; 450 College of 1

Pharmacy South, University of Georgia Athens, GA 30602, 2

Phone: 706-542-3792 Fax: 706-542-5358 E-Mail: briansc@uga.edu Keywords: Drug resistance, Lipids, Lipidomics, Lipid metabolism, Lipid species, Mass Spectrometry, Metastasis, Prostate Cancer, Prostate gland 1 Abstract Background The association of circulating lipids with clinical outcomes of drug-resistant castration-resistant prostate cancer (DR-CRPC) is not fully understood. While it is known that increases in select lipids correlate to decreased survival, neither the mechanisms mediating these alterations nor the correlation of resistance to drug treatments is well characterized. Methods This gap-in-knowledge was addressed using in vitro models of non-cancerous, hormone-sensitive, CRPC and drug-resistant cell lines
